# Multiple DNA marker-assisted diversity analysis of Indian mango (*Mangifera indica* L.) populations

**DOI:** 10.1038/s41598-021-89470-3

**Published:** 2021-05-14

**Authors:** Ram Chandra Jena, Pradeep Kumar Chand

**Affiliations:** Plant Biotechnology Laboratory, Post-Graduate Department of Botany, Utkal University, VaniVihar, Bhubaneswar, Odisha 751004 India

**Keywords:** Biotechnology, Ecology, Molecular biology, Plant sciences, Ecology

## Abstract

Arbitrary (65 RAPD, 25 ISSR, 23 DAMD), gene-targeted (22 SCoT, 33 CBDP) and co-dominant sequence specific (40 SSR) markers were used individually, or in combinations, to examine the genetic variability within and among 70 selected Indian mango genotypes based on geographic origin (East India, West India, North India, South India) and fruit status (Selection, Hybrid, Landrace). The highest genetic variability was demonstrated by the East Indian populations, followed by those from South India, West India, and North India, when measured in terms of Na, Ne, H, I, PB%, Ht and Hs. Interestingly, the local genotypes of Odisha, which forms a part of East Indian populations, showed the highest diversity compared to hybrid or selection groups, suggesting that the indigenous genotypes hold a greater potential for exploiting the unique and favourable alleles. The maximum genetic variability was detected in geographical/fruit status populations with SSRs (Na—1.76/1.88, Ne—1.48/1.51, H—0.28/0.30, I—0.41/0.45, PB%—76.1/86.9, Ht—0.31/0.32 and Hs—0.28/0.30), followed by CBDPs and SCoTs, reflecting their preeminence for examining the level of genetic polymorphism and diversity. Outcome of AMOVA based analyses as well as low-to-moderate coefficient of genetic differentiation (Gst) and high gene flow (Nm) indicated a greater amount of intra-population genetic variation compared to heterogeneity at inter-population level. Information generated through this investigation could facilitate conservation and further exploitation of mango germplasm including genetic improvement through breeding.

## Introduction

Mango (*Mangifera indica* L., Family: Anacardiaceae, Order: Sapindales), is one the most important fruit crops, and referred to as ‘King of fruits’ in the tropical world^1^. Endowed with the largest mango gene pool around the globe^2–5^, with a high degree of diversity, India is considered to be the centre of origin of mango from where it is believed to have spread progressively to tropical and subtropical provinces of the world. India's eco-geographic diversity has been responsible for a good number of fruit crop type; no wonder that India ranks second in global fruits production, next to China^6^. The leading mango producing States of India are Uttar Pradesh (23.06%), Andhra Pradesh (16.06%), Karnataka (9.29%), Telengana (8.54%), Bihar (7.51%), Gujarat (6.30%), Tamil Nadu (5.87%), West Bengal (4.24%) and Odisha (4.14%)^7^. The wild as well as cultivated forms of mangoes (Local, Hybrid and Selection) in India exhibit a wide diversity of fruit forms, flavour, size and taste^8^.Unfortunately, at present, only a meagre number of about 20 landraces are commercially cultivated, majority of them being restricted to specific regions^9^.

Basal to species diversity is the genetic diversity, which is considered as a key precursor in studying a species, because the magnitude and range of heterogeneity in populations of such species influence its evolutionary potential^10^. Determining genetic diversity is critically useful so that elite genotypes are identified, multiplied and conserved^11^. Conservation of mango genetic resources is crucial to the long-term survival, sustainable production and genetic improvement of the commercially profitable genotypes. Assessment of the genetic diversity level and genetic structure of a populations of a species helps ascertain its current status and threats; thus, could provide a basis for adopting appropriate scientific management policies and devising effective conservation strategies^12^. It is essential to preserve genetic diversity to promote adaptability of the populations to changing environment as well as to preserve a large gene pool for future genetic improvement^13^, the latter achievable through either conventional hybridization or molecular breeding. Knowledge about the genetic background of the parents is a necessary prelude to develop new varieties endowed with superior fruit features and more adapted to the changing climatic conditions. It is also important to assess the phylogenetic interrelationships among cultivated relatives. Local elites, being superior performer genotypes, within the same region sharing the same climatic conditions can be ideal candidates for breeding purposes compared to those available in geographically isolated populations at long distances^14^. True-to-type propagation for multiplication and conservation of the natural population of mangoes is imperative to preserve the existing diversity in the local population, avert imminent extinction of the elite genotypes available in these localities, and to reduce the risk of loss of desirable features (such as fruit quality, for mangoes) owing to uncontrolled natural outbreeding depression, if any^15^. The application of reliable and rapid DNA-based diagnostic techniques to discriminate/relate established genotypes and unexplored/under-explored local ones (landraces) is rewarding to improve the efficacy of genotype management for use in breeding and conservation of fruit tree species for production of certified plant material with superior fruit quality^16^. Besides, documentation in form of molecular atlas, as DNA fingerprints, generated for elite mango genotypes and local landraces hold significance in context of ascertaining genetic authenticity (trueness-to-type) of the conserved germplasm.

Information on the extent and structure of genetic variation in germplasm collections is essential for the effective conservation, efficient management and prospective utilization of biodiversity in any crop species. In addition, knowledge of the genetic and population diversity of germplasm collections serves a solid foundation for crop improvement. It is essential to first define the population diversity within the germplasm to avoid spurious associations while performing association mapping studies^17^. Population diversity is widely used in conservation biology to quantify relationships and differences among populations^18^.

Determination of the extent of genetic diversity in fruit crop populations is essential to guide strategies for their conservation and sustainable utilization for genetic improvement. Estimating and understanding of the levels of variability existing within and between the populations do not only facilitate formulation of appropriate conservation strategies, as it reflects the status and survival potential of populations, but also it helps to resolve ecological, taxonomic, phylogenetic and demographic questions of great relevance^19,20^. Conservation of genetic diversity is extremely vital for the long-term subsistence of a plant species, because loss of genetic variability within populations may significantly decrease adaptability to environmental alteration and increase of disappearance risk^21^. Understanding the relative significance of specific processes, such as inbreeding, gene flow, genetic drift, and selection that structure diversity within and among populations can deliver means to evaluate future risk of erosion of diversity and to design effective conservation approaches for rare taxa^22^. Disappearance of a single population would remove any distinctive biological traits that it retains and might eventually lead to varietal elimination^21^. George et al.^23^ suggested that examining the extent of genetic diversity within and among populations can help in understanding evolutionary contrivances, such as genetic drift and can serve as an indicator of the extent of gene flow and population divergence.

The use of DNA-based markers offers a potential strategy for genetic analysis at population level. DNA markers are not influenced by environmental conditions and, therefore, can be used to help describe patterns of genetic variation among plant populations^24,25^. Different PCR-based DNA marker techniques are available for studying plant population genetics. These may be classified as arbitrarily amplified DNA markers, DNA sequence based markers and gene targeted functional markers. Molecular methods differ from each other with respect to important features such as genomic abundance, level of polymorphism detected, locus specificity, reproducibility, technical requirements and cost^26^. Assessment of genetic diversity within and among population have been reported in horticultural crops, such as wild apricot^27^, bottle gourd^28^, apple^29^ and in other plants, including Christmas orchids^30,31^, mungbean^32^, *Pinus nigra*^33^, * Populus wulianensis*^34^ etc. to understand spatial and temporal differences between populations. Yet, studies on Indian mangoes are limited to the cultivars of Andhra Pradesh (a State of India) and use of SSR markers alone^35^. However, no studies have looked at population diversity in a diverse collection of mangoes in India using a range of DNA fingerprinting techniques such as RAPD, ISSR, DAMD, SCoT, CBDP and SSR markers, neither individually nor cumulatively. Comparative studies of different molecular techniques for measuring population diversity have already been performed in apricot^36^, *Ocimum*^37^, bamboo^38^, mungbean^32^, etc. However, only a few reports exist pertaining to multiple marker comparison for genetic diversity in mangoes, particularly those of Thailand, Vietnam, and China^39^ and of Gir forest region of India^40^. The present investigation is a maiden effort to assess genetic variability within and among populations with geographic affiliation (East India, West India, North India and South India) and those based on fruit status (selection, hybrid, indigenous), encompassing 70 Indian mango genotypes, employing individual and combination of arbitrary (RAPD, ISSR, DAMD), gene-targeted (SCoT, CBDP) and sequence specific marker systems (SSR). Our work was aimed at following objectives: i) to compare the efficacy of various PCR-based markers (used either individually or cumulatively) for analysing genetic diversity among and within populations of Indian mangoes from various geographical locations and fruit status background. ii) to investigate population differentiation based on genetic diversity parameters for further use in population and conservation genetics. iii) to estimate the gene flow among populations.

## Results

### Individual and cumulative DNA marker-based banding statistics

Of a total of 80 RAPD primers subject to initial screening, 65 primers yielded clear and reproducible patterns of bands ranging in size from 100 to 3000 bp with an average of 15.91 bands per primer. Twenty-five different ISSR primers yielded clear and bright bands of sizes ranging 100–2500 bp, with an average of 18.04 bands per primer (Table 2). Initial screening of 41 DAMD primers identified 23 primers with an average of 16.57 bands per primer. Of 48 different SCoT primers tested 22 were able to produce clear and bright bands of sizes, which ranged between 100–3000 bp with an average of 17.64 bands per primer. A total of 56 CBDP primers were screened, of which 33 primers generated distinct and reproducible banding patterns of different band lengths (100–3000 bp), with an average of 16.03 bands per primer. Of a total 72 SSR primers screened, features and evaluation details resulting from 40 scorable SSR primer pairs were considerably informative. All the tested loci were polymorphic with an average of 4.13 alleles/locus. The representative banding patterns of 70 mango genotypes using OPA 18 (RAPD), ISSR-9 (ISSR), HBV (DAMD), SCoT 8 (SCoT), CAAT-3 (CBDP) and SSR-20 (SSR) primer are given in supplementary online files (Suppl. Figure 1a-f). For cumulative analysis, a total of 113 arbitrary primers (65 RAPD, 25 ISSR and 23 DAMD) were employed, which generated 16.84 average no. of bands/primer across all mango genotypes examined. The combined gene targeted markers (SCoT + CBDP) resulted 16.83 average number of bands/primer. A total of 208 primers yielded 12.60 average bands per primer among all genotypes of mangoes under investigation (Tables 1, 2).

**Table 1:** Indian mango genotypes of 4 Geographical Populations (East India, West India, North India, South India) and 3 Fruit Status-based Populations (Selection, Hybrid, Landrace) audited.

Sl. no	Genotype and its geographic distribution		Fruit status	Sl. no	Genotype and its geographic distribution		Fruit status
1	Suvernarekha	East India	Selection	36	Raspuri	West India	Selection
2	Fazli		Selection	37	Ratna		Hybrid
3	Gulab Khas		Selection	38	Kesar		Selection
4	Lal Sundari		Selection	39	Niranjan		Selection
5	Sabri		Hybrid	40	Sai Sugandh		Hybrid
6	Mahmood Bahar		Hybrid	41	Alphonso		Selection
7	Alfazli		Hybrid	42	Sindhu		Hybrid
8	Bileimundei		Landrace	43	Amrapali	North India	Hybrid
9	Dophasal		Landrace	44	Pusa Surya		Hybrid
10	Miskanta		Landrace	45	Chausa		Selection
11	KanchaMitha		Landrace	46	Langra		Selection
12	Jamuna		Landrace	47	Bombay Green		Selection
13	Karpura Bhog		Landrace	48	Ambika		Hybrid
14	Hunkagaja		Landrace	49	Mallika		Hybrid
15	Lajkuli Bandana		Landrace	50	Dasahari		Selection
16	Khajara		Landrace	51	Pusa Arunima		Hybrid
17	Special Phatansa		Landrace	52	Amin Tehasil		Selection
18	Ganga		Landrace	53	Sundar Pasand		Selection
19	Hatimundi		Landrace	54	Dasheri Clone51		Hybrid
20	Baldev		Landrace	55	Prabha Shankar		Hybrid
21	Kuanri		Landrace	56	ArkaNeelkiran	South India	Hybrid
22	Premsagar		Landrace	57	PKM-2		Hybrid
23	Anandsagar		Landrace	58	Manjeera		Hybrid
24	Shreemati		Landrace	59	ArkaPuneet		Hybrid
25	Baramasi		Landrace	60	Totapuri		Selection
26	Khandagiri		Landrace	61	Totapuri Red Small		Selection
27	Aishwarya		Landrace	62	Jehangir		Selection
28	Hamilton Sundari		Landrace	63	Janardhan Pasand		Selection
29	Bathua		Selection	64	Banganapalli		Selection
30	Rajapuri	West India	Selection	65	Neelum		Selection
31	Neeleshan Gujarat		Hybrid	66	Mulgoa		Selection
32	Vanraj		Selection	67	Arka Anmol		Hybrid
33	Neeleshwari		Hybrid	68	Arka Aruna		Hybrid
34	Pairi		Selection	69	Cherukrasam		Selection
35	Neelphonso		Hybrid	70	PKM-1		Hybrid

**Table 2:** Estimates of comparative performance of individual and cumulative DNA marker techniques applied to selected Indian mango genotypes.

Sl. no.	Marker (s)	Number of primers screened	No. of primers used	Average no. of bands/primer	Size range of bands (bp)
1	RAPD	80	65	15.91	100–3000
2	ISSR	53	25	18.04	100–2500
3	DAMD	41	23	16.57	100–3000
4	SCoT	48	22	17.64	100–3000
5	CBDP	56	33	16.03	100–3000
6	SSR	72	40	4.13	45–530
7	RAPD + ISSR + DAMD	174	113	16.84	100–3000
8	SCoT + CBDP	104	55	16.83	100–3000
Total of Independent Markers		350	208	12.60	45–3000

### Genetic variability within and among populations and gene flow

#### Variability across geographical populations

Based on their geographical origin, 70 mango genotypes were grouped into four populations—East India (EI), West India (WI), North India (NI), and South India (SI) (Table 1). Additionally, these genotypes were clubbed into three different populations based on their fruit status as Selection (S), Hybrid (H) and, Local (L) (Table 1). Comparative performances of marker systems used were evaluated to determine the utility of each method either individually or in combination for diversity studies in mango populations based on different criteria which are presented in Tables 2 and 3. The genetic variability parameters, such as the number of alleles (Na), effective number of alleles (Ne), Nei’s genetic diversity (h), Shannon’s information index (I), polymorphic band percentage (PB%), total genetic diversity (Ht), genetic diversity within population (Hs), coefficient of genetic differentiation (Gst) and gene flow (Nm) using different marker systems, either individually or in combination, to assess the genetic variability are presented in Table 3.

**Table 3:** Mean genetic variability statistics among 4 Geographical Populations (East India, West India, North India, South India) and 3 Fruit Status Populations (S = Selection, H = Hybrid and L = Landrace) of selected Indian mangoes based on individual and cumulative DNA marker analysis.

Marker (s)	Population^a^	Na	Ne	H	I	PB (%)	Ht		Hs		Gst		Nm
RAPD	EI (29)	1.88 ± 0.32	1.42 ± 0.34	0.25 ± 0.17	0.39 ± 0.23	88.05							
	WI (13)	1.54 ± 0.49	1.34 ± 0.36	0.20 ± 0.19	0.30 ± 0.28	53.78							
	NI (13)	1.55 ± 0.49	1.33 ± 0.36	0.20 ± 0.19	0.30 ± 0.28	54.98							
	SI (15)	1.75 ± 0.43	1.40 ± 0.34	0.24 ± 0.18	0.37 ± 0.25	75.30							
	Mean	1.68 ± 0.43	1.37 ± 0.35	0.23 ± 0.18	0.34 ± 0.24	68.03	0.26 ± 0.03		0.22 ± 0.02		0.140		3.071
	S (26)	1.81 ± 0.39	1.42 ± 0.35	0.25 ± 0.18	0.28 ± 0.24	80.88							
	H (23)	1.76 ± 0.42	1.39 ± 0.34	0.24 ± 0.17	0.37 ± 0.25	76.10							
	L (21)	1.84 ± 0.36	1.42 ± 0.35	0.25 ± 0.17	0.39 ± 0.24	84.46							
	Mean	1.80 ± 0.39	1.41 ± 0.35	0.25 ± 0.17	0.35 ± 0.24	80.48	0.27 ± 0.02		0.25 ± 0.02		0.115		3.821
ISSR	EI (29)	1.86 ± 0.35	1.45 ± 0.35	0.27 ± 0.18	0.40 ± 0.24	85.63							
	WI (13)	1.57 ± 0.49	1.34 ± 0.34	0.20 ± 0.19	0.31 ± 0.27	57.47							
	NI (13)	1.52 ± 0.50	1.30 ± 0.34	0.18 ± 0.19	0.28 ± 0.28	51.72							
	SI (15)	1.71 ± 0.45	1.42 ± 0.36	0.25 ± 0.19	0.37 ± 0.27	70.69							
	Mean	1.66 ± 0.45	1.38 ± 0.35	0.23 ± 0.18	0.34 ± 0.26	66.337	0.27 ± 0.03	0.23 ± 0.02		0.104		4.298	
	S (26)	1.80 ± 0.40	1.44 ± 0.36	0.26 ± 0.18	0.39 ± 0.25	79.89							
	H (23)	1.71 ± 0.45	1.42 ± 0.37	0.25 ± 0.19	0.37 ± 0.27	71.26							
	L (21)	1.82 ± 0.38	1.45 ± 0.36	0.26 ± 0.18	0.40 ± 0.24	82.18							
	Mean	1.78 ± 0.41	1.44 ± 0.36	0.26 ± 0.18	0.39 ± 0.25	77.78	0.27 ± 0.03	0.26 ± 0.02		0.061		7.654	
DAMD	EI (29)	1.84 ± 0.36	1.40 ± 0.35	0.24 ± 0.17	0.37 ± 0.25	84.31							
	WI (13)	1.55 ± 0.50	1.33 ± 0.34	0.20 ± 0.19	0.30 ± 0.28	54.90							
	NI (13)	1.49 ± 0.50	1.28 ± 0.34	0.17 ± 0.19	0.25 ± 0.27	49.02							
	SI (15)	1.74 ± 0.44	1.35 ± 0.35	0.21 ± 0.18	0.33 ± 0.25	74.51							
	Mean	1.66 ± 0.45	1.34 ± 0.35	0.20 ± 0.18	0.31 ± 0.26	65.69	0.23 ± 0.03		0.20 ± 0.02		0.150		2.836
	S (26)	1.69 ± 0.46	1.35 ± 0.31	0.22 ± 0.17	0.34 ± 0.25	68.63							
	H (23)	1.74 ± 0.44	1.34 ± 0.35	0.21 ± 0.18	0.32 ± 0.25	74.51							
	L (21)	1.80 ± 0.40	1.40 ± 0.36	0.24 ± 0.18	0.37 ± 0.25	80.39							
	Mean	1.74 ± 0.43	1.36 ± 0.34	0.22 ± 0.18	0.34 ± 0.25	74.51	0.24 ± 0.03		0.22 ± 0.02		0.065		7.194
SCoT	EI (29)	1.83 ± 0.37	1.44 ± 0.36	0.26 ± 0.18	0.39 ± 0.25	83.10							
	WI (13)	1.56 ± 0.49	1.35 ± 0.35	0.21 ± 0.19	0.31 ± 0.28	56.34							
	NI (13)	1.62 ± 0.48	1.41 ± 0.39	0.23 ± 0.20	0.34 ± 0.29	61.97							
	SI (15)	1.84 ± 0.36	1.47 ± 0.34	0.28 ± 0.17	0.43 ± 0.23	84.51							
	Mean	1.71 ± 0.43	1.42 ± 0.36	0.25 ± 0.19	0.37 ± 0.26	71.48	0.27 ± 0.03		0.25 ± 0.02		0.100		4.494
	S (26)	1.75 ± 0.43	1.44 ± 0.38	0.25 ± 0.19	0.38 ± 0.27	74.65							
	H (23)	1.86 ± 0.35	1.46 ± 0.34	0.28 ± 0.17	0.42 ± 0.23	85.92							
	L (21)	1.80 ± 0.40	1.43 ± 0.35	0.26 ± 0.18	0.39 ± 0.25	80.28							
	Mean	1.80 ± 0.39	1.45 ± 0.34	0.26 ± 0.18	0.40 ± 0.25	80.28	0.28 ± 0.03		0.26 ± 0.02		0.053		8.909
CBDP	EI (29)	1.92 ± 0.27	1.47 ± 0.33	0.28 ± 0.16	0.43 ± 0.21	92.14							
	WI (13)	1.57 ± 0.49	1.38 ± 0.38	0.22 ± 0.20	0.33 ± 0.29	57.14							
	NI (13)	1.67 ± 0.47	1.39 ± 0.35	0.23 ± 0.18	0.35 ± 0.26	67.14							
	SI (15)	1.80 ± 0.40	1.44 ± 0.35	0.26 ± 0.17	0.40 ± 0.24	80.00							
	Mean	1.74 ± 0.40	1.42 ± 0.35	0.25 ± 0.18	0.38 ± 0.25	74.10	0.28 ± 0.02		0.25 ± 0.02		0.102		4.402
	S (26)	1.84 ± 0.36	1.44 ± 0.36	0.26 ± 0.17	0.40 ± 0.23	84.29							
	H (23)	1.82 ± 0.38	1.44 ± 0.35	0.27 ± 0.17	0.41 ± 0.24	82.14							
	L (21)	1.86 ± 0.35	1.48 ± 0.34	0.28 ± 0.17	0.43 ± 0.23	85.71							
	Mean	1.87 ± 0.34	1.46 ± 0.34	0.27 ± 0.17	0.41 ± 0.23	84.05	0.29 ± 0.02		0.27 ± 0.02		0.054		8.710
SSR	EI (29)	1.91 ± 0.38	1.58 ± 0.38	0.32 ± 0.19	0.47 ± 0.26	91.35							
	WI (13)	1.65 ± 0.48	1.42 ± 0.36	0.25 ± 0.19	0.37 ± 0.28	65.22							
	NI (13)	1.65 ± 0.48	1.40 ± 0.36	0.24 ± 0.19	0.36 ± 0.28	65.22							
	SI (15)	1.83 ± 0.28	1.49 ± 0.36	0.29 ± 0.17	0.44 ± 0.22	82.61							
	Mean	1.76 ± 0.41	1.48 ± 0.36	0.28 ± 0.19	0.41 ± 0.26	76.1	0.31 ± 0.02		0.28 ± 0.02		0.156		2.714
	S (26)	1.87 ± 0.34	1.49 ± 0.33	0.29 ± 0.17	0.44 ± 0.23	86.96							
	H (23)	1.83 ± 0.28	1.48 ± 0.35	0.29 ± 0.16	0.44 ± 0.21	82.61							
	L (21)	1.91 ± 0.38	1.57 ± 0.38	0.32 ± 0.19	0.47 ± 0.26	91.30							
	Mean	1.88 ± 0.36	1.51 ± 0.35	0.30 ± 0.17	0.45 ± 0.23	86.95	0.32 ± 0.02		0.30 ± 0.02		0.070		6.566
RAPD + ISSR + DAMD	EI (29)	1.87 ± 0.33	1.43 ± 0.35	0.26 ± 0.17	0.40 ± 0.23	86.76							
	WI (13)	1.55 ± 0.49	1.34 ± 0.35	0.20 ± 0.19	0.30 ± 0.28	55.25							
	NI (13)	1.53 ± 0.49	1.32 ± 0.35	0.19 ± 0.19	0.28 ± 0.28	53.15							
	SI (15)	1.73 ± 0.44	1.40 ± 0.35	0.24 ± 0.18	0.37 ± 0.25	73.53							
	Mean	1.67 ± 0.44	1.37 ± 0.35	0.22 ± 0.18	0.34 ± 0.26	67.62	0.26 ± 0.03		0.22 ± 0.02		0.147		2.907
	S (26)	1.80 ± 0.40	1.41 ± 0.35	0.25 ± 0.18	0.38 ± 0.25	79.20							
	H (23)	1.74 ± 0.43	1.40 ± 0.35	0.24 ± 0.18	0.36 ± 0.25	74.16							
	L (21)	1.83 ± 0.37	1.43 ± 0.35	0.26 ± 0.18	0.39 ± 0.24	83.19							
	Mean	1.79 ± 0.40	1.42 ± 0.35	0.25 ± 0.18	0.38 ± 0.25	78.85	0.27 ± 0.03		0.24 ± 0.02		0.078		5.893
SCoT + CBDP	EI (29)	1.89 ± 0.31	1.46 ± 0.34	0.28 ± 0.17	0.42 ± 0.23	89.10							
	WI (13)	1.57 ± 0.49	1.37 ± 0.37	0.22 ± 0.20	0.32 ± 0.29	56.87							
	NI (13)	1.65 ± 0.47	1.40 ± 0.36	0.23 ± 0.19	0.35 ± 0.27	65.40							
	SI (15)	1.81 ± 0.38	1.45 ± 0.34	0.27 ± 0.17	0.40 ± 0.24	81.52							
	Mean	1.73 ± 0.41	1.42 ± 0.35	0.25 ± 0.18	0.38 ± 0.26	73.22	0.28 ± 0.02		0.25 ± 0.02		0.101		4.432
	S(26)	1.81 ± 0.39	1.44 ± 0.35	0.26 ± 0.18	0.39 ± 0.24	81.04							
	H (23)	1.83 ± 0.37	1.45 ± 0.34	0.27 ± 0.17	0.41 ± 0.23	83.41							
	L (21)	1.84 ± 0.36	1.46 ± 0.35	0.28 ± 0.17	0.42 ± 0.24	83.89							
	Mean	1.83 ± 0.37	1.45 ± 0.35	0.27 ± 0.17	0.41 ± 0.24	82.78	0.28 ± 0.02		0.27 ± 0.02		0.053		8.774

*Na* observed number of alleles, *Ne* effective number of alleles, *H* Nei’s genetic diversity, *I* Shannon’s information index, *NPB* number of polymorphic bands, PB (%): polymorphic band percentage, *Ht* total genetic diversity, *Hs* genetic diversity within population, *Gst* coefficient of genetic differentiation, *Nm* Gene flow.

^a^The numbers in parenthesis in each population denote the number of genotypes from that population used in the present study.

Co-dominant SSR markers were able to detect highest level of variability, which was demonstrated by the highest mean values of Na (1.76), Ne (1.48), H (0.28), I (0.41) and PB% (76.1), Ht (0.31) and Hs (0.28) compared to other markers. On average, CBDP and SCoT markers closely followed SSR in terms of Na (1.74, 1.71), Ne (1.42), H (0.25), I (0.38, 0.37), PB% (74.10, 71.48), Ht (0.28, 0.27) and Hs (0.25). The RAPD, ISSR and DAMD markers exhibited a comparatively lower mean values for genetic diversity indices as compared to the other markers. Interestingly, Na was the highest (1.68) for RAPD markers among arbitrary markers while it was at par for ISSR and DAMD (1.66).The polymorphic band percentage (PB%) was also higher for RAPD (68.03%) compared to ISSR (66.33%) and DAMD (65.69%). Values for mean effective number of alleles (Ne), total genetic diversity (Ht) and genetic diversity within population (Hs) found to be more for ISSR (1.38, 0.27, 0.23) followed by RAPD (1.37, 0.26, 0.22) and DAMD (1.34, 0.23, 0.20) respectively, while other two crucial genetic diversity parameters, such as Nei’s genetic diversity (H) and mean Shannon’s information index (I) was unvaryingly higher for ISSR and RAPD (0.23,0.34) compared to DAMD (0.20, 0.31). Genetic structure of geographical/fruit status populations was also measured using combination of various arbitrary markers (RAPD + ISSR + DAMD) and gene targeted markers (SCoT + CBDP); the latter had superior entries for genetic diversity parameters, (Na = 1.73, Ne = 1.42, H = 0.25, I = 0.38, PB = 73.22%, Ht = 0.28 and Hs = 0.25) in comparison with the former (Na = 1.67, Ne = 1.37, H = 0.22, I = 0.34, PB = 67.62%, Ht = 0.26 and Hs = 0.22). Hence, the gene targeted markers were more efficient than arbitrary markers for evaluating genetic diversity of different populations. The different diversity parameters indicated that Indian mango germplasm hold a considerably high level of genetic diversity. Among all the markers tested with four different populations, the estimator of population substructure or coefficient of genetic differentiation (Gst) value were more with SSR and DAMD (0.156 and 0.150) indicating that about 15–16% of the total genetic variability resided among the population. These Gst values were associated harmoniously with less levels of gene flow (Nm), of 2.714 and 2.836 with SSR and DAMD, respectively compared to other markers. These Gst and Nm were also varied for RAPD (0.140, 3.071), ISSR (0.104, 4.298), SCoT (0.10, 4.494) and CBDP (0.102, 4.402) markers respectively (Table 3). Mean coefficient of gene differentiation (Gst) value 0.147 indicated that about 15% genetic diversity resided among the populations as revealed by arbitrary markers, which was higher than the diversity informed by gene targeted markers (Gst—0.101). Gene flow estimate by gene targeted markers (SCoT + CBDP) was found to be high (4.432), relative to that of arbitrary markers (RAPD + ISSR + DAMD) (2.907) (Table 3).

Among the four geographic populations, mango genotypes of East India (EI) exhibited the maximum variability. The extent of genetic variability level for East India population was measured in respect of several parameters, such as observed number of alleles (Na), effective number of alleles (Ne), Nei’s genetic diversity (h), Shannon’s information index (I) and percent polymorphism (PB %). Data recorded varied with RAPD (1.88, 1.42, 0.25, 0.39 and 88.05%), ISSR (1.86, 1.45, 0.27, 0.40 and 85.63%), for DAMD (1.84, 1.39, 0.24, 0.37 and 84.31%), CBDP (1.92, 1.47, 0.28, 0.43 and 92.14%), SSR (1.91, 1.58, 0.32, 0.47 and 91.35%), RAPD + ISSR + DAMD (1.87, 1.43, 0.26, 0.40 and 87.76%), and SCoT + CBDP (1.89, 1.46, 0.28, 0.42 and 89.10%), respectively (Table 3). The only exception was SCoT marker which detected the highest diversities for South India population (PB% = 84.51, Na = 1.84, Ne = 1.47, H = 0.28 and I = 0.43). Among the different markers used, all these parameters were recorded highest for SSR (PB% and Na), while CBDP revealed the highest polymorphism level (92.14%) and number of alleles (1.92). The South Indian cultivars also exhibited high value diversity parameters with RAPD (H = 0.24, I = 0.37, PPB = 75.30%), ISSR (H = 0.25, I = 0.37, PPB = 70.69%), DAMD (H = 0.21, I = 0.33, PPB = 74.51%), CBDP (H = 0.26, I = 0.40, PPB = 80%) and SSR (H = 0.29, I = 0.41, PPB = 82.61%) markers. The North Indian and South Indian genotypes showed the moderate values for genetic diversity indices. The observed numbers of alleles were higher than that effective in all populations. Presence of private alleles i.e. number of alleles unique to a single population were observed, the most being generated for East Indian (EI) (3–16) followed by South Indian (SI) (3–6) populations.

#### Variability across fruit status populations

Population analysis performed on the fruit status unveiled noticeable outcomes. The intra-population genetic diversity study revealed the highest values of Na (1.84, 1.82, 1.80, 1.86, 1.91), Ne (1.42, 1.45, 1.40, 1.48, 1.57), H (0.25, 0.26, 0.24, 0.28, 0.32), I (0.39, 0.40, 0.37, 0.43, 0.47) and PB% (84.46%, 82.18%, 80.39%, 85.71%, 82.61%) by RAPD, ISSR, DAMD, CBDP and SSR markers correspondingly among the genotypes of local population (L). The exception was SCoT marker which uncovered highest Na (1.86), Ne (1.46), H (0.28), I (0.42) and PB% (85.92%) among the genotypes of hybrid population (H) (Table 3). The combined marker techniques, SCoT + CBDP as well as RAPD, ISSR + DAMD also experienced similar pattern for values of Na (1.84, 1.83), Ne (1.46, 1.43), H (0.28, 0.26), I (0.42, 0.39) and PB% (83.89%, 83.19%), with the highest value observed in genotypes of local population (L).

The population of Selection genotypes (S) exhibited higher variability than that of hybrids (H) (Na = 1.80 / 1.71, Ne = 1.44 / 1.42, H = 0.26 / 0.25, I = 0.39 / 0.37, PB% = 79.89 / 71.26) as noticed by ISSR markers. The SSR and CBDP markers experienced a similar trend in terms of variability quantities for selection and hybrid populations. Interestingly, CBDP and SSR markers had higher mean values of Na (1.87, 1.88) across all the populations compared to SCoT and RAPD (1.80), ISSR (1.78) and DAMD (1.74). Average values for other parameters like Ne (1.51), H (0.29), I (0.45), PB% (86.95), Ht (0.32) and Hs (0.30) were highest for SSR. The gene targeted markers CBDP and SCoT had Ne (1.45, 1.44), H (0.27, 0.26), I (0.41, 0.40), PB% (84.05, 80.28), Ht (0.28, 0.27) and Hs (0.27, 0.26), respectively. Considering different arbitrary markers, the mean effective number of alleles (Ne), Nei’s genetic diversity (H), Shannon’s information index (I) and total genetic diversity (Ht) was found to be maximum for ISSR (1.43, 0.26, 0.39, 0.27), followed by RAPD (1.41, 0.25, 0.35, 0.27) and DAMD (1.36, 0.22, 0.34, 0.23), respectively.

Higher PB% values were found in RAPD (80.48) followed by ISSR (77.78) and DAMD (74.51), while another critical genetic diversity parameter, i.e. Hs (genetic diversity within population) was equally higher for ISSR and RAPD (0.26, 0.25) compared to DAMD (0.22). Gene targeted markers had higher levels of diversity in terms of average Na (1.83), Ne (1.45), H (0.27), I (0.41), PB% (82.78), Ht (0.28) and Hs (0.27) than those from arbitrary ones Na (1.79), Ne (1.41), H (0.25), I (0.38), PB% (78.85), Ht (0.27) and Hs (0.25). Gst value was the highest in RAPD (0.115) followed by SSR (0.07), DAMD (0.065), ISSR (0.061), CBDP (0.054) and SCoT (0.053). This indicated the superior ability of the RAPD markers to evaluate the variation among local, hybrid and selection genotypes, whilst the trend for gene flow (Nm) among the populations was the reverse, being the highest for SCoT (8.91) followed by CBDP (8.71), ISSR (7.65), DAMD (7.19), SSR (6.56) and RAPD (3.82).

### Analysis of molecular variance (AMOVA)

#### Variability across geographical populations

**Figure 1:**
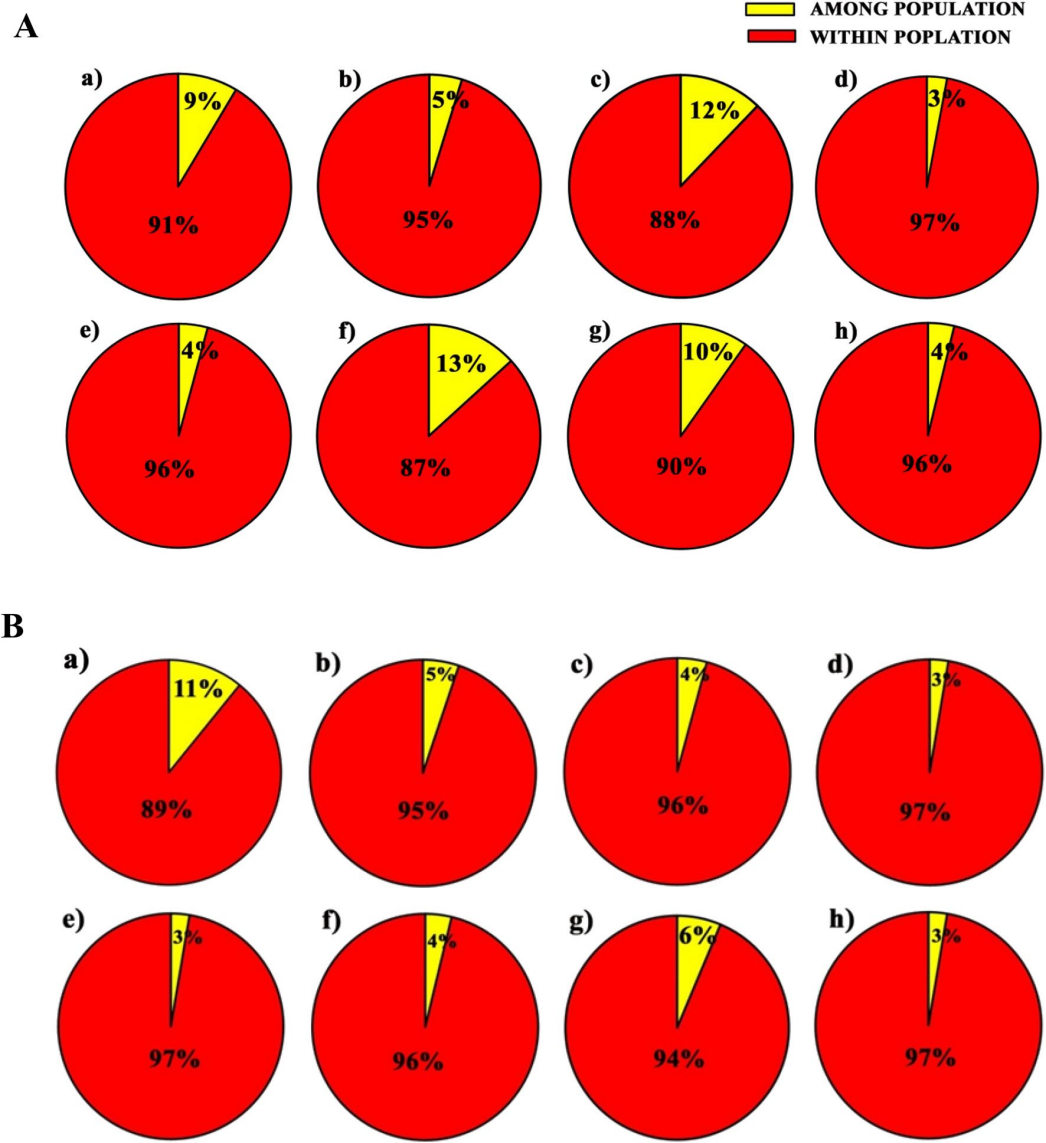
AMOVA analysis of (**A**) 4 Geographical Populations (East India = EI, West India = WI, North India = NI, South India = SI) and (**B**) Fruit Status Populations (S = Selection, H = Hybrid and L = Landrace) of selected Indian mangoes based on individual and cumulative DNA marker systems: (**a**) RAPD (**b**) ISSR (**c**) DAMD, (**d**) SCoT (**e**) CBDP (**f**) SSR (**g**) RAPD + ISSR + DAMD (h) SCoT + CBDP.

Hierarchical AMOVA study was performed to estimate population differentiation among selected mango genotypes. The present study with different six marker systems showed that geographic variation was partitioning between 3–13% and maximum variation was present within geographic populations (87–97%). RAPD data showed 9% variation partitioned among geographic populations, whereas 91% variation was existent within the geographic populations (Fig. 1Aa, Supplementary Table S1). Similarly, ISSR based AMOVA analysis showed 5% and 95% variation among and within populations, respectively (Fig. 1Ab, Supplementary Table S1). DAMD marker based AMOVA analysis accounted for 12% variation among geographic populations and 88% variation within geographical populations (Fig. 1Ac). SCoT marker based AMOVA analysis showed the least variation among populations (3%) and maximum variation (97%) within populations (Fig. 1Ad).With CBDP marker, 4% variation was accumulated within populations while between populations it was 96% (Fig. 1Ae, Supplementary Table S1). Of all the six marker systems used for AMOVA analysis, SSR marker based analysis showed maximum variation among populations (13%) (Fig. 1Af, Supplementary Table S1). The overall ΦPT values for RAPD, ISSR, DAMD, SCoT, CBDP and SSR were 0.086, 0.047, 0.117, 0.029, 0.042 and 0.122, respectively. Comparison of variations between the combination of the markers in AMOVA demonstrated a higher variation within the populations by SCoT + CBDP (96%) and RAPD + ISSR + DAMD (90%), while a lower amount of variation among the populations was observed by them (4% and 10%; Φ_PT_—0.038 and 0.098 ) (Figs. 1Ag–h, Supplementary Table S1).

**Figure 2:**
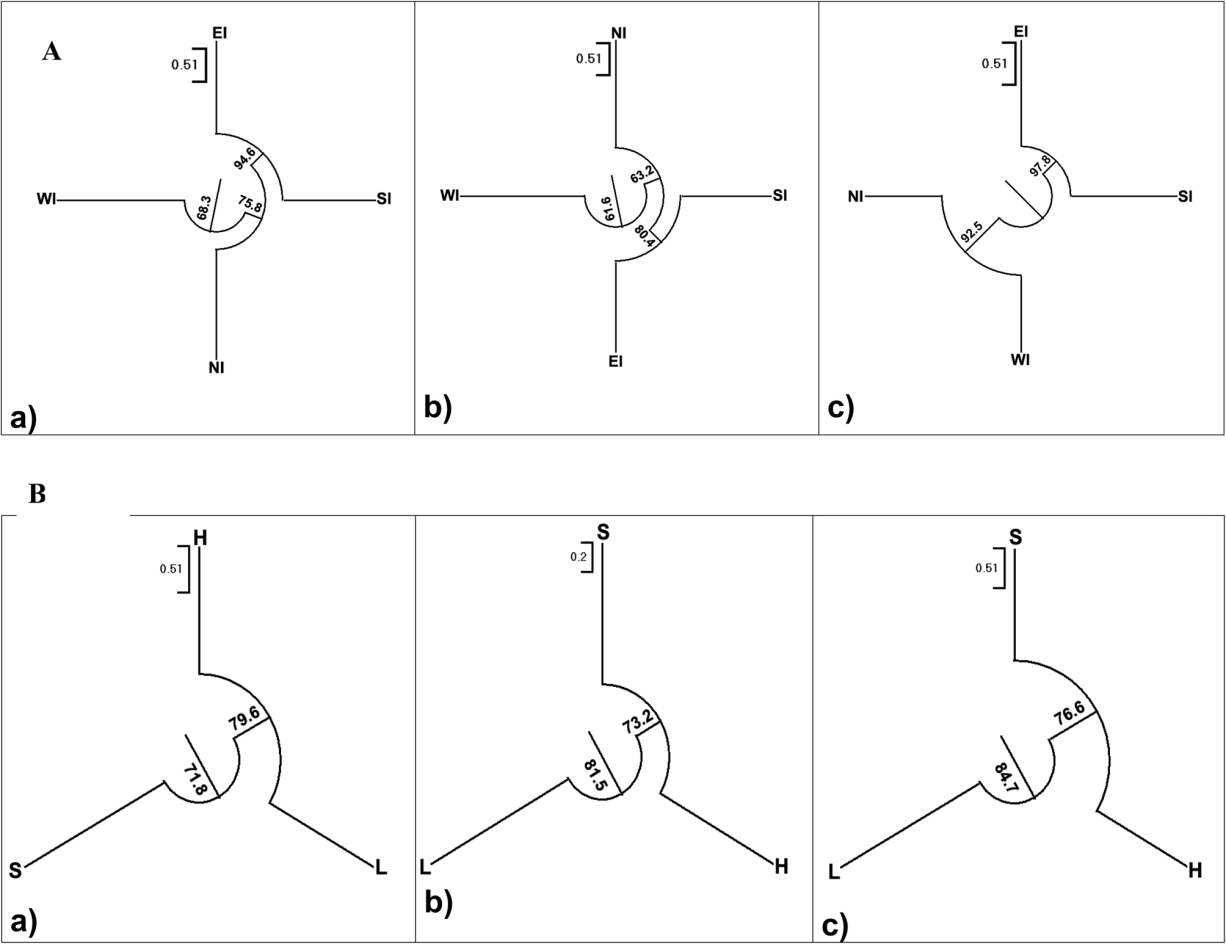
Neighbour joining dendrogram for (**A**) 4 different Geographic Populations (East India = EI , West India = WI, North India = NI, South India = SI) and (**B**) three different Fruit Status Populations (S = Selection, H = Hybrid and L = Landrace) of selected Indian mangoes, based on Nei’s genetic distance using DNA marker systems: (**a**) cumulative Arbitrary (RAPD + ISSR + DAMD), (**b**) cumulative Gene targeted (SCoT + CBDP) (**c**) SSR.

#### Variability across fruit status populations

The present investigation developed an alternative AMOVA model with populations nested within local, hybrid and selection genotypes. This showed that 3–11% of variance was segregating among populations and 89–97% within populations taking into account all the marker systems. Data depicted that majority of variations were significant related to differences among genotypes within populations with respect to RAPDs (89%), ISSRs (95%), DAMDs (96%), SCoTs (97%), CBDPs (97%) and SSRs (96%) while among population variance components were very low i.e. 11% (RAPD), 5% (ISSR), 4% (DAMD, SSR), 3% (SCoT, CBDP). Examining the overall Φ_PT_ values for RAPD, ISSR, DAMD, SCoT, CBDP and SSR they were found to be 0.084, 0.052, 0.042, 0.027, 0.028 and 0.039, respectively (Fig. 1Ba-f, Supplementary Table S2). AMOVA based on cumulative arbitrary markers presented a similar trend and about 6% variation among the populations and 94% variation within population were revealed (Fig. 1Bg, Supplementary Table S2). Cumulative gene targeted marker based analysis demonstrated a maximum 97% variation within populations and 3% among populations used for AMOVA analysis (Fig. 1Bh, Supplementary Table S2).

### Genetic similarity and cluster analysis among populations

#### Geographical populations

To illustrate further the phylogenetic relationship between four different geographic populations, the Neighbor-joining method was employed to construct the phylogenetic tree (NJ dendrogram) of SSR, cumulative RAPD + ISSR + DAMD and SCoT + CBDP markers (Supplementary Table S3, Fig. 2Aa-c). To illustrate the phylogenetic relationship among individual 70 mango genotypes, a dendrogram was plotted using the Jaccard’s similarity coefficient of cumulative RAPD + ISSR + DAMD + SCoT + CBDP + SSR markers profile, depicted in supplementary file (Suppl. Figure 2a). A similar pattern was observed in the grouping of genotypes of 70 Indian mango genotypes in 2D and 3D PCA depicted in supplementary files (Suppl. Figure 2b & c). The Nei’s genetic distance and genetic identity both were calculated and represented in below and above diagonals of the Supplementary Table S3. The Nei’s genetic identity ranged from 0.83 to 0.93 for RAPD + ISSR + DAMD, 0.90 to 0.95 for SCoT + CBDP and 0.81 to 0.94 for SSR. The genetic relationship between EI and SI populations was the closest in respect of all the three methods (SSR—0.94, SCoT + CBDP—0.95, RAPD + ISSR + DAMD—0.93), hence they clustered together. For SSR markers, The NJ dendrogram depicted that EI and SI populations (0.94) were grouped together as did the populations of WI and NI (0.91), while the farthest genetic identity (0.81) was between EI and WI populations. In RAPD + ISSR + DAMD based dendrogram, the genotypes of NI population seemed to be closely related to SI and EI pair (NI *vs* SI—0.91; NI *vs* EI—0.90), while genotypes from WI [WI *vs* EI (0.83)/SI (0.88)/NI (0.90)] are slightly distinct from the three populations. The same pattern of clustering was also manifested with SCoT + CBDP, but the identity values were more pronounced (SI *vs* NI—0.93; EI vs NI—0.92; EI vs WI—0.91, NI vs WI—0.91 and SI vs WI—0.90).

#### Fruit status populations

Similarly, Neighbor-joining method was employed to construct the phylogenetic tree (NJ dendrogram) among three fruit status populations (L, H, S) using SSR, cumulative RAPD + ISSR + DAMD and SCoT + CBDP markers (Supplementary Table S4, Fig. 2Ba-c). The Nei’s genetic identity ranged 0.91–0.96 for RAPD + ISSR + DAMD, 0.94–0.97 for SCoT + CBDP and 0.92–0.96 for SSR. The genetic identity between Hybrid (H) and Selection (S) populations was maximum for all the three methods (SSR—0.96, SCoT + CBDP—0.97, RAPD + ISSR + DAMD—0.96) having the closest genetic relationship. In SCoT + CBDP based dendrogram, the hybrid population (H) seemed to be closely related to the selection population (S) (0.97), while the local population ‘L’ [L *vs* S (0.94) / H (0.94)] was distinct from the two populations. The same pattern of clustering was also manifested with SSR but the identity values were more pronounced (L *vs* S—0.92 / H—0.92). For RAPD + ISSR + DAMD markers, ‘H’ depicted maximum genetic identity with both populations ‘S’ and ‘L’ (0.96). The NJ dendrogram depicted that ‘H’ and ‘S’ populations were grouped together, while the farthest genetic identity was 0.91 between ‘L’ and ‘S’ populations.

## Discussion

The maintenance of genetic variation is a key objective for conservation^41^. Focal to guiding strategies for conservation and sustainable exploitation of crops for genetic improvement is the determination of genetic diversity within and among populations. Based on the genetic variability parameters viz. Na, Ne, h, I, PB%, Ht, Hs, GST and Nm results of the current study on various marker systems differed according to mango populations based on geographic origin and fruit status. The genetic diversity indices like percent polymorphism, observed number of alleles, effective number of alleles, Nei’s genetic diversity, Shannon's index reflects diversity and differentiation among the germplasm collections and reveal genetic diversity within and between the populations. The higher the indices, greater is the genetic diversity.

Among the mango germplasm collected from four different geographical regions, the highest genetic diversity was observed in East Indian population (EI), followed by that of South India (SI) > West India (WI) > North India (NI), suggesting that mango genotypes from the eastern and southern parts of India possess relatively higher genetic variation and has a greater capability to acclimatize and evolve than the other populations. The observed number of alleles were higher than that effective in all populations. Similar kind of observations were reported in other tree species like Apricot^42^ and *Zanthoxylum Spp.*^15^.The primary reasons for differences in genetic variation can be attributed to climate conditions^43^, topography^44^, mating system and seed dispersal method^45^. Genetic diversity is often positively associated with population size; larger populations generally hold proportionately higher levels of genetic diversity. While studying different apricot cultivars of North China, Li et al.^46^ also emphasized that the genetic diversity was affected by the population size and a larger sample size would generate more accurate results. In our case, the reasons for the high level of genetic diversity observed in EI may be attributed to potential genetic status, eco-geographical factors, advantageous tropical growing conditions, wide geographic distribution and population size. Interestingly, most of the East Indian genotypes are indigenous hence as expected, the local genotypes generally showed more diversity than hybrid or selection groups genotypes in population analysis based on fruit status. This may be due to the presence of unique alleles present in these indigenous populations, which have been lost during prolonged cultivation over the years or long-term adoption, etc. in case of hybrid and selection populations. The presence of ‘private alleles’ in different populations represent a unique source of genetic diversity in the studied Indian mango germplasm. East Indian and South Indian population had the highest numbers of these private alleles, as detected using all the six marker systems, i.e. RAPD (16, 6), ISSR (12, 3), SCoT (3, 5), DAMD (8, 6), CBDP (8, 2) and SSR (8, 4). The private alleles can be used as molecular signatures (ID marks) in fingerprinting studies and such data could be used in assessment of genetic purity of the populations. Most of the local populations encompass indigenous genotypes of Odisha State and these results demonstrate the potential of local germplasm for exploiting the unique and favourable alleles present therein for mango germplasm improvement programmes.Hence, such information will aid the selection of cultivars for germplasm conservation and implementation in modern-day mango breeding, by providing information on diverse genetic backgrounds in native and local genotypes.

The existing variations in the nature of genotypes or group of genotypes can be identified using a specific statistical method or combination of methods^47^. The selection of a particular type of molecular marker is important and critically depends on the intended use^48^. Sivaprakash et al.^49^ suggested that the ability of a marker system to resolve genetic variation may be directly related to the degree of polymorphism. Polymorphism in a given population is often due to the existence of genetic variants represented by the number of alleles at a locus and their frequency of distribution in a population. Comparison of RAPD, ISSR, DAMD, SSR, SCoT and CBDP analysis in respect of diversity parameters showed a substantial level of genetic variation and broad genetic base in studied mango populations. Genetic variability among Indian mangoes selected for geographical and fruit status population analysis in the present study, as estimated by parameters such as Na, Ne, h, I, PB%, Ht and Hs, were found to be the highest with SSRs, followed by gene targeted markers like CBDPs and SCoTs, thus reflecting the preeminence of the former one at levels of genetic The polymorphic band percentage (PB%) was also established to be higher in both geographical and fruit status populations for RAPD (68.03, 80.48) compared to ISSR (66.33, 77.78) and DAMD (65.69, 74.51). The reason for higher genetic diversity revealed by SSR markers may be attributable to their multi-allelic nature, high polymorphism and information content as well as unique mechanism responsible for generating SSR allelic diversity by replication slippage. Nei’s genetic diversity, Shannon’s index, means values of effective number of alleles, observed number of allele and Percent polymorphism were also reported high in trees like *Zanthoxylum spp.*^15^, *Punica granatum* L.^50^ and *Lagenaria siceraria*^28^ using different markers.

The high levels of polymorphism obtained with different markers for geographical (> 65%) and fruit status (> 74%) populations clearly proved their usefulness in the genetic variability studies on Indian mango populations. Variation in the polymorphism in marker systems detected by different primers may be attributed to the fact that their specificity and efficiency are governed by different nucleotide sequence. The gene targeted and microsatellite markers generated abundant polymorphism, thus they could be applied to identify the plant materials having close relationships in different populations. In addition to polymorphism detection, their higher scores of Na, Ne, H, I, PB%, Ht and Hs than other studied marker systems, were perhaps because of their derivation from genic regions of the genome.

The genetic differentiation of a species reflects the interactions of various evolutionary processes including long-term evolutionary history, such as shifts in distribution, habitat fragmentation and population isolation, mutation, genetic drift, mating system, gene flow and natural selection^51^. Various parameters viz. geographical isolation, population fragmentation, breeding system and genetic drifts may be responsible for the high population differentiation^52^. The coefficient of gene differentiation (Gst) indicates the level of differentiation according to the scale, low (0.00–0.05), moderate (0.06–0.15), high (0.16–0.25) or very high (> 0.25)^53^. Gst derived gene flow (Nm) is another way to ascertain genetic variability to a population which can affect the genetic structure too. It allows members of one gene pool to mate with members of another gene pool, leading to a shift of the allele frequencies and decrease in the degree of population differentiation^54^. Consequently, more evident the gene flow, the lower is the degree of genetic differentiation. Estimate of genetic flow (Nm) has been classified as low (Nm < 1), moderate (Nm > 1) and extensive (Nm > 4) in population genetics^55^. Nm values greater than one is strong enough to prevent substantial differentiation due to genetic drift^56^. Geographic origin based populations were moderately structured by all the six marker systems, individually and in combination, due to Gst value of 0.10–0.15 and an overall Gst-derived estimate of gene flow of 2.71- 4.49. The moderate to extensive gene flow among the populations signifies a substantial extent of gene exchange between various populations. Mean coefficient of gene differentiation (Gst) value 0.147 indicated that about 15% genetic diversity resided among the species by arbitrary markers (RAPD + ISSR + DAMD), which is higher than the diversity informed by gene targeted markers (Gst—0.101). Gene flow estimate by gene targeted markers was found to be high (4.432) and it was lower than the gene flow estimate of arbitrary markers (2.907). These Gst and Nm were also varied for RAPD (0.140, 3.071), ISSR (0.104, 4.298), SCoT (0.10, 4.494) and CBDP (0.102, 4.402) markers, respectively. Such differentiation is in contrast to observations in cowpea (Gst = 0.409;^57^. The Gst and Nm ranges obtained in the present study using different marker types were comparable to the values (Gst : 0.087–0.180, Nm : 2.278–5.240) recorded for 46 different accessions of a wild plant, *Amygdalusmira* examined using SSR, ISSR and SSR + ISSR markers^58^.

Investigation of an alternative population exploration based on fruit status unfolded a low-to-moderate extent of genetic differentiation and high gene flow (Gst—0.053–0.115, Nm—3.82–8.90). Gst value was highest in RAPD (0.115), followed by SSR (0.07), DAMD (0.065), ISSR (0.061), CAAT (0.054) and SCoT (0.053), which indicate the highest ability of the RAPD markers to evaluate the variation among local, hybrid and selection genotypes, whilst the trend for gene flow (Nm) among the populations was reversed, being the maximum for SCoT (8.91) followed by CBDP (8.71), ISSR (7.65), DAMD (7.19), SSR (6.56) and RAPD (3.82). Low levels of Gst and high Nm were also obtained for bottle gourd^28^ similar to the present study. SSR and DAMD markers reflected slightly more differentiating ability depending on geography, so did the RAPDs for local, hybrid and selection populations.

These results were further supported by the AMOVA analysis which revealed that maximum (> 87%) of the total genetic diversity by all six marker systems was distributed within populations, whereas a less fraction (3–13%) of the diversity was attributed to differences among populations. Similar patterns of more genetic variation within populations were demonstrated in other perennial species; for instance, Fig. ^59^, strawberry^60^, mulberry^61^ and Koehne^58^, using different marker systems. SSR and DAMD was partitioning maximum (13% and 12%) among geographic populations, whereas RAPD was partitioning maximum variation of 11% among local, hybrid and selection populations. Similar to our results, was AMOVA analysis of 19 varieties of *Ficuscarica*^49^, which showed SSR to represent 9.82% group variation, higher compared to RAPD (0.71%), ISSR (6.69%) and RAPD + ISSR (2.59%). The overall Φ_PT_ values confirming geographic distribution had little effect on genetic diversity of mango genotypes. This study clearly indicates frequent exchange of gene pool among the populations perhaps mediated by rampant cross-pollination.

The levels of genetic diversity and the distribution of variability within and between plant populations have generally been interpreted as the result of the balanced combination of the reproductive system and the history of the species^62^. Perennial and allogamous species typically exhibit higher levels of genetic diversity within, rather than between, in contrast to inbreeding or selfing annual species populations, thereby indicating the influence of biological characteristics on these parameters^63^. Previous reports also revealed that self-pollinating species have relatively less within-population genetic variation than out-crossing species^64^. Nm may be greater in outcrossing species because the pollen dispersal mechanisms which are much more developed in perennial out-crossers than in inbreds or partially self-pollinated herbaceous plants. Furthermore, the geographic distribution of a species is also highly dependent on the type of pollinators. The potential factors like widespread nature, pollination by insects may have contributed to this scattered pattern of genetic divergence leading to less population differentiation, high gene flow, and frequent gene exchange. The results of the present study suggested that more genetic variation of the species could be captured when sampling a larger number of plants from each population or geographic region. Previous studies also demonstrated that gene mutation, gene flow, population size, sampling strategy can influence genetic variation^65^.

Nei’s genetic distances and identity were estimated for determining the relationships among mangoes from the four geographic populations *i.e.* East India (EI), West India (WI), North India (NI) and South India (SI) and a dendrogram was generated. Results revealed minimum values of Nei’s genetic diversity (h) distances between EI and SI populations which were 0.07, 0.05, 0.06 for cumulative gene targeted, arbitrary and SSR markers, respectively. These two populations are neighbours to each other and hence geographical closeness might have played a role in the clustering of the provenances. Being so, both the regions share similar climatic conditions i.e. tropical and sub-tropical. However another distinctive factor contributing to such proximity in genetic diversity might be due to the latitude, longitude and altitude of the respective regions. These above mentioned factors are found to be responsible in studies with other plant species (Xie et al.^43^, Yan et al.^44^). Compared to EI and SI, varying climatic conditions (ranges from tropical wet and dry to semi-arid) might have resulted in distinctive genetic variability in WI population in respect of all DNA marker systems employed. The same pattern of clustering among geographical populations was also manifested with SSR, cumulative RAPD + ISSR + DAMD and SCoT + CBDP markers. In the present study the genetic variability is found to be congruent with the geographical diversity. Likewise, NJ dendrogram was prepared among three fruit status populations. The NJ clustering revealed more or less similar grouping pattern for all the three methods. While Hybrid (H) and Selection (S) populations revealed closest genetic relationship, the local (L) population was distinctive.

These results were supported by population analysis of cumulative arbitrary markers, of which RAPD revealed the most genetic differentiation (Gst—0.115, less gene flow (Nm—3.821) and maximum variations among population (11%) in respect of determining fruit status. Aros et al.^66^ established UPGMA cluster analysis among wild species and cultivated varieties of *alstroemeria* (Peruvian lily) through RAPD markers, comparable to our study. Discordance between dendrograms obtained by diverse marker types could be explained by different nature of each markers (genetically inert and functionally active), different regions coverage of the genome targeted by different marker techniques, extent of polymorphism perceived and the number of loci^67,68^. High bootstrap values at most of the nodes supported the stability of these dendrograms. Co-phenetic analysis was also done to evaluate the ‘goodness of fit’ of the resulting phylogenetic trees. Data based on RAPD + ISSR + DAMD, SCoT + CBDP and ISSR markers revealed the consistency of the inferred relationships as the co-phenetic correlation value obtained were ≥ 0.7 for all marker systems and indicated a good fit.

In the present investigation, comparisons were made between use of combinational markers, such as arbitrary dominant (RAPD + ISSR + DAMD) and gene targeted (SCoT + CBDP) type, for testing the combined ability of the markers for genetic diversity assessment among mango populations of India. Cumulative multi-marker analyses have been reported to be more informative than individual ones applied to horticultural crops like papaya^69^ and jatropha^70^. Our studies have shown that SCoT + CBDP markers were more effective than RAPD + ISSR + DAMD markers in estimating diversity among geographical and fruit status populations. A comparative account of the gene targeted and neutral markers in detecting genetic variations in *Morinda tomentosa* was studied by Arya et al.^61^, who reported that gene targeted markers were more useful than random markers in detecting polymorphism, similar to our results. It is, therefore, advisable to employ combined and multiple marker analysis (distinct and analogous) while performing analytical assessment of genetic diversity and relationship among populations, because such markers target to different genomic fractions, thus providing complementary information and offering more accurate and conclusive results^71^.

Results of our investigation demonstrate that while within population the genetic diversity was high, among populations it was relatively low in magnitude. The level of genetic variation is influenced by an array of determinants, such as gene flow, geographic conditions and genetic drift. Geographic isolation may lead to a loss of genetic diversity due to reduced gene flow and genetic drift. In absence of timely steps as protective measures, the intra-population genetic diversity might decline due to inbreeding influence. Hence, genetic rescue of promising local mango landraces via in situ and ex situ conservation measures would be highly desirable^12^. The present study using multiple DNA marker-based diagnostic profiles of mangoes provide the means of rapid characterization of established as well as local genotypes within the populations and, thus, enable the selection of appropriate genotypes for conservation, sustainable management and commercial exploration, such as profitable utilization in bio-prospection programs and also as parents for breeding aimed at genetic improvement of this important edible plant genetic resource.

## Conclusions

In essence, the present study demonstrates the usefulness of DNA marker systems for elucidation of the genetic variability within and among Indian mango populations based on their geographic origin or status of the fruit.

The maximum genetic heterogeneity perceived for East Indian populations of mango genotypes, including local elites of the State of Odisha, indicates that they hold the highest proficiency to adapt and evolve compared to other geographical populations. SSR and gene targeted markers were more efficient than arbitrary markers for evaluating genetic diversity of different populations. SSR and DAMD markers reflected slightly more differentiating ability among geographic populations, so did the RAPDs for local, hybrid and selection populations.The high levels of polymorphism obtained with all marker systems used in the present study for population diversity analysis clearly proved their usefulness in estimating the genetic variability among Indian mango germplasm. Combined marker approach was more informative than use of individual markers in population diversity analysis. SCoT + CBDP markers proved to be more effective than RAPD + ISSR + DAMD markers with respect to parameters for evaluating diversity among populations grouped based on geographical locations or fruit status. Understanding the genetic diversity among and within populations of Indian mangoes will be a useful resource in respect of future germplasm conservation, maintenance, exploitation and breeding success, aimed at their genetic improvement.

## Materials and methods

### Plant material

In the present investigation, we have used 70 promising Indian mango genotypes (Table 1), encompassing 26 selections (commercially grown mango genotypes), 23 hybrids (developed through breeding at different institutes) and 21 local germplasms (landraces), representing different eco-geographical locations of India and are maintained in field gene banks.

### Genomic DNA extraction and quantification

Fresh and preserved leaves of three different ages (tender, young and mature) of 70 mango genotypes were used for DNA extraction. Extraction of total genomic DNA was carried out as described by Doyle and Doyle^72,73^ with minor modifications^8^. A sharp glowing band was observed indicating the presence of good quality genomic DNA. By comparing the fluorescent intensity of the bands with the standard (λ DNA/ *EcoRI* digest, Bangalore Genei Pvt. Ltd., India), DNA concentration was estimated following the method described by Sambrook et al.^74^. Parts of stock DNA samples were diluted with appropriate amount of TE buffer to yield a working concentration of 29–40 ng/µl for downstream marker analysis. DNA samples were stored in a refrigerator (4 °C).

### PCR amplification

Genotyping was performed using a total of 278 primers that included individual and combination of arbitrary (80 RAPD, 53 ISSR, 41 DAMD), gene-targeted (48 SCoT, 56 CBDP) and co-dominant sequence specific (72 SSR) markers using the protocol described by Jena et al.^8^. Only 208 primers out of the 40 primers screened, resulted in discrete profiles consisting of polymorphic reproducible fragments were validated for further analysis (Table 2).The RAPD primers include OPA, OPC, OPD (Operon Technologies, Alameda, California, USA) and RPI-C Series (Bangalore Genei, India). ISSR primers (University of British Columbia, Canada). DAMD primers were selected basing on the minisatellite core sequences in rice^75–77^, humans^78,79^, fungi^80^ and phage M13;SCoT primers (designed by Collard et al.^81^; Luo et al.^82^). A total of 56 CBDP primers (50 designed and 6 from Singh et al. ^83^) and microsatellite primers (SSR) selected from earlier reports^84–87^ were synthesized by Bangalore Genei, India. Detailed Primer sequence information of all markers are documented (Jena^8^). All amplified products (RAPD, ISSR, DAMD, SCoT and CBDP) were loaded in wells and resolved on 1.5% agarose gel in 1 × TBE buffer by electrophoresis. For SSR, amplification products were resolved on 3% agarose gels. The amplified fragments were photographed using gel documentation system (Bio-Rad, USA) and stored as digital images using the built-in software.

### Molecular data analysis

All amplifications were repeated thrice and only reproducible and consistent bands were considered for analysis. The distinct amplicons were scored visually as discrete variables using 1 for presence and 0 for absence separately for each marker and a binary matrix was obtained. Binary marker data were used for the purpose of revealing association of molecular markers with pomometric traits.

### Analysis of population genetic variability and AMOVA

To evaluate variability among and within populations, 70 different mango genotypes were classified based on:

- Geographic origin [(i) East India (EI, 29 genotypes), (ii) West India (WI, 13 genotypes) (iii) North India (NI, 13 genotypes), and (iv) South India (SI, 15 genotypes)]
- Fruit status [(i) Selection (S, 26 genotypes), (ii) Hybrid (H, 23 genotypes) and (iii) Local (L, 21 genotypes)]

The basic parameters for genetic diversity such as observed number of alleles (Na), effective number of alleles (Ne), Nei's genetic diversity (H), Shannon's information index (I), polymorphic bands percentage (PB%), total genetic diversity (Ht), genetic diversity within population (Hs), coefficient of genetic differentiation (Gst) and gene flow (Nm) were calculated using POPGENE software version 1.31^88^. Genetic diversity within and among populations was estimated by the method of analysis of molecular variance (AMOVA) using GenAlEx 6.502 software^89^ for studying molecular variations at population level. The significance of the results was tested using 9999 random permutations of the data.

### Similarity-based clustering and construction of phylogenetic tree

Pairwise-similarity matrices were generated by calculating Jaccard's similarity coefficient^90^ among all possible pairs to accomplish genetic similarity between the genotypes with the SIMQUAL (Similarity of Qualitative Data) option of Numerical Taxonomy and Multivariate Analysis System, NTSYS-pc software version 2.02^91^. These similarity matrices were then run on Sequential, Agglomerative, Hierarchical and Nested (SAHN) clustering method and subjected for construction of dendrogram by the unweighted pair group method with arithmetic average (UPGMA)^92^ clustering algorithm with NTSYS-pc. To evaluate the relationship among different populations of Indian mangoes a neighbour joining (NJ) dendrogram was constructed based on Nei's genetic distance^93^. To estimate the robustness and validity of dendrogram typology and clustering bootstrap analyses were performed of 1000 bootstrap samples using the software WINBOOT^94^.

## Supplementary Information

Supplementary Information.

## References

[1] Purseglove JW (1972). Mangoes west of India. Acta Hortic..

[2] Mukherjee SK (1953). Origin, distribution and phylogenetic affinities of the species of *Mangifera indica* L. Bot. J. Linn. Soc..

[3] Kostermans AJGH, Bompard JM (1993). The Mangoes: Their Botany, Nomenclature.

[4] Ravishankar KV, Lalitha A, Anand L, Dinesh MR (2000). Assessment of genetic relatedness among mango cultivars of India using RAPD markers. J. Hortic. Sci. Biotech..

[5] Karihaloo JL, Dwivedi YK, Archak S, Gaikwad AB (2003). Analysis of genetic diversity of Indian mango cultivars using RAPD markers. J. Hortic. Sci. Biotech..

[6] APEDA, The Agricultural and Processed Food Products Export Development Authority http://apeda.gov.in/apedawebsite/sixheadproduct/FFV.htm (2017).

[7] National Horticultural Board, Ministry of Agriculture and Farmers Welfare Government of India 85, Institutional Area, Sector-18, Gurugram 122015 (Haryana), India http://www.nhb.gov.in (2016-17).

[8] Jena, R.C. DNA fingerprinting of some promising Indian genotypes and hybrids of mango (*Mangifera indica* L.). PhD Thesis (pp 1–422). Utkal University, India (2019).

[9] Yadav IS, Rajan S (1993). Genetic resources of mango. Adv. Hortic..

[10] Zhang J (2015). Potential of start codon targeted (SCoT) markers to estimate genetic diversity and relationships among Chinese *Elymus sibiricus* accessions. Molecules.

[11] Harisaranraj R, Prasitha R, Saravana Babu S, Suresh K (2008). Analysis of inter-species relationships of Ocimum species using RAPD markers. Ethnobotanical Leaflets..

[12] Liu, H. *et al*. Genetic diversity and population structure of the endangered plant Salix taishanensis based on CDDP markers. *Glob Ecol. Conserv*. 24, (2020).

[13] Mahar KS (2013). Estimation of genetic variability and population structure in *Sapindus trifoliatus* L., using DNA fingerprinting methods. Trees.

[14] Kalpana D (2012). Assessment of genetic diversity among varieties of mulberry using RAPD and ISSR fingerprinting. Sci. Hortic..

[15] Medhi K (2014). High gene flow and genetic diversity in three economically important Zanthoxylum Spp. of Upper Brahmaputra Valley Zone of NE India using molecular markers. Meta Gene..

[16] Wunsch A, Hormaza JI (2002). Cultivar identification and genetic fingerprinting of temperate fruit tree species using DNA markers. Euphytica.

[17] Flint-Garcia SA (2005). Maize association population: a high-resolution platform for quantitative trait locus dissection. Plant J..

[18] Breton C, Pinatel C, Medail F, Bonhomme F, Berville A (2008). Comparison between classical and Bayesian methods to investigate the history of olive cultivars using SSR-polymorphisms. Plant Sci..

[19] Pillon Y, Qamaruz-Zaman F, Fay MF, Hendoux F, Piquot Y (2007). Genetic diversity and ecological differentiation in the endangered fen orchid (*Liparis loeselii*). Conserv. Genet..

[20] Mahar KS, Rana TS, Ranade SA, Meena B (2011). Genetic variability and population structure in *Sapindus emarginatus* Vahl from India. Gene.

[21] Izawa T, Kawahara T, Takahashi H (2007). Genetic diversity of an endangered plant, *Cypripedium macranthos*var. rebunense (Orchidaceae): Background genetic research for future conservation. Conserv. Genet..

[22] Neel MC, Ellstrand NC (2003). Conservation of genetic diversity in the endangered plant *Eriogonum ovalifolium* var. *vineum* (Polygonaceae). Conserv. Genet..

[23] George S, Sharma J, Yadon VL (2009). Genetic diversity of the endangered and narrow endemic *Piperia yadonii* (Orchidaceae) assessed with ISSR polymorphisms. Am. J. Bot..

[24] Marsjan, P. & Oldenbroek, J.K. Molecular markers, a tool for exploring genetic diversity. The State of the World’s Animal Genetic Resources for Food and Agriculture, (pp. 359–379). FAO Research report, Rome (2007).

[25] Kumar P, Gupta VK, Misra AK, Modi DR, Pandey BK (2009). Potential of molecular markers in plant biotechnology. Plant Omics..

[26] Agarwal M, Shrivastava N, Padh H (2008). Advances in molecular marker techniques and their applications in plant sciences. Plant Cell Rep..

[27] Li M, Zhao Z, Miao XJ (2013). Genetic variability of wild apricot (*Prunus Armeniaca* L.) populations in the Ili Valley as revealed by ISSR markers. Genet. Resour. Crop Evol..

[28] Abdin MZ (2014). Population structure and genetic diversity in bottle gourd [*Lagenaria siceraria* (Mol.) Standl.] germplasm from India assessed by ISSR marker. Plant Syst. Evol..

[29] Fazeli S, Sheidai M, Farahani F, Noormohammadi Z (2016). Looking for genetic diversity in Iranian apple cultivars (*Malus* × *domestica* Borkh.). J Sci..

[30] Qian X, Wang C, Tian M (2013). Genetic diversity and population differentiation of *Calanthe tsoongiana*, a rare and endemic orchid in China. Int J Mol Sci..

[31] Singh N (2013). Comparison of SSR and SNP markers in estimation of genetic diversity and population structure of Indian rice varieties. PLoS ONE.

[32] Jena RC, Agarwal K, Ghosh TS, Chand PK (2017). Population structuring of selected mungbean landraces of the Odisha State of India via DNA marker-based genetic diversity analysis. Agric. Gene..

[33] Dias A (2020). Portuguese *Pinus nigra* JF Arnold populations: genetic diversity, structure and relationships inferred by SSR markers. Ann. For. Sci..

[34] Wu Q, Zang F, Ma Y, Zheng Y, Zang D (2020). Analysis of genetic diversity and population structure in endangered *Populus wulianensis* based on 18 newly developed EST-SSR markers. Glob. Ecol. Conserv..

[35] Surapaneni M (2013). Population structure and genetic analysis of different utility types of mango (*Mangifera indica* L.) germplasm of Andhra Pradesh state of India using microsatellite markers. Plant Syst. Evol..

[36] Yilmaz KU, Paydas-Kargi S, Dogan Y, Kafkas S (2012). Genetic diversity analysis based on ISSR, RAPD and SSR among Turkish apricot germplasms in Iran Caucasian eco-geographical group. Sci. Hortic..

[37] Patel HK, Fougat RS, Kumar S, Mistry JG, Kumar M (2015). Detection of genetic variation in *Ocimum* species using RAPD and ISSR markers. 3. Biotech.

[38] Desai P (2015). Comparative assessment of genetic diversity among Indian bamboo genotypes using RAPD and ISSR markers. Mol. Biol. Rep..

[39] Luo C (2011). Genetic diversity of mango cultivars estimated using SCoT and ISSR markers. Biochem. Syst. Ecol..

[40] Gajera HP, Tomar RS, Patel SV, Viradia RR, Golakiya BA (2011). Comparison of RAPD and ISSR markers for genetic diversity analysis among different endangered *Mangifera indica* genotypes of Indian Gir forest region. J. Plant Biochem. Biotech..

[41] Hamrick, J. L. & Godt, M. J. W. Conservation genetics of endemic plant species. In Avise, J. C., & J. L. Hamrick (Eds.), Conservation genetics: case histories from nature. (pp. 281–30). Chapman and Hall, New York (1996).

[42] Wang W, Li Z, Li Y (2014). Isolation and characterization of microsatellite markers for *Cotinus coggygria**Scop*. (*Anacardiaceae*) by 454 pyro sequencing. Molecules.

[43] Xie WG, Zhang XQ, Ma X, Huang LK, Zeng B (2009). Genetic variation of *Dactylis glomerata* germplasm from Southwest China detected by SSR markers. Acta Pratacult..

[44] Yan XB, Guo YX, Zhou H, Wang K (2006). Analysis of geographical conditions affected on genetic variation and relationship among populations of *Elymus*. J. Plant Res. Environ..

[45] Hamrick JL, Godt MJW, Sherman-Broyles SL (1992). Factors influencing levels of genetic diversity in plant species. New For..

[46] Li M, Zhao Z, Miao X (2014). Genetic diversity and relationships of apricot cultivars in North China revealed by ISSR and SRAP markers. Sci. Hortic..

[47] Kubik C, Honig J, Meyer WA, Stacy AB (2009). Genetic diversity of creeping bent-grass cultivars using SSR markers. Int. Turfgrass Soc. Res. J..

[48] Gupta PK, Roy JK (2002). Molecular markers in crop improvement: Present status and future needs in India. Plant Cell Tiss. Org..

[49] Sivaprakash KR, Prasanth SR, Mohanty BP, Parida A (2004). Genetic diversity of black gram landraces as evaluated by AFLP markers. Curr. Sci..

[50] Noormohammadi Z (2012). Genetic Variation among Iranian Pomegranates (*Punica granatum* L.) using RAPD, ISSR and SSR Markers. Aust. J. Crop Sci..

[51] Schaal BA, Hayworth DA, Olsen KM, Rauscher JT, Smith WA (1998). Phylogeographic studies in plants: problems and prospects. Mol. Ecol..

[52] Zong M (2008). Genetic diversity in geographic differentiation in the threatened species *Dysosma pleiantha* in China as revealed by ISSR analysis. Biochem. Genet..

[53] Wright S (1978). Evolution and the Genetics of Population.

[54] Slatin M (1987). Gene flow and geographic structure of natural populations. Science.

[55] Kumar A, Mishra P, Singh SC, Sundaresan V (2014). Efficiency of ISSR and RAPD markers in genetic divergence analysis and conservation management of *Justicia adhatoda* L., a medicinal plant. Plant Syst. Evol..

[56] Slatkin M, Barton NH (1989). A comparison of three indirect methods for estimating the average level of gene flow. Evolution.

[57] Kouam EB, Pasquet RS, Elteraifi I, Muluvi GM (2011). Genetic diversity and population structure of *Vigna unguiculata* ssp. *unguiculata* var. spontanea in Sudan. J. Res. Biol..

[58] Xing C, Tian Y, Meng F (2015). Evaluation of genetic diversity in *Amygdalus mira* (Koehne) Ricker using SSR and ISSR markers. Plant Syst. Evol..

[59] Ikegami H, Nogata H, Hirashima K, Awamura M, Nakahara T (2009). Analysis of genetic diversity among European and Asian fig varieties (*Ficus carica* L.) using ISSR, RAPD, and SSR markers. Genet. Resour. Crop Evol..

[60] Takrouni MM, Boussaid M (2010). Genetic diversity and population's structure in Tunisian strawberry tree (*Arbutus undo* L.). Sci. Hortic..

[61] Arya L, Narayanan RK, Verma M, Singh AK, Gupta V (2014). Genetic diversity and population structure analyses of *Morinda**tomentosa* Heyne, with neutral and gene based markers. Genet. Resour. Crop Evol..

[62] Hamrick, J. L., Godt, M. J. W., Murawski, D. A., & Loveless, M. D. Correlations between species traits and allozyme diversity: Implications for conservation biology. In Falk, D.A.S., & K. E. Holsinger (Eds.), Genetics and conservation of rare plants. (pp. 75–86), Oxford University Press, Oxford (1991).

[63] Loveless MD, Hamrick JL (1984). Ecological determinants of genetic structure in plant populations. Annu. Rev. Ecol. Evol. Syst..

[64] Schoen DJ, Brown AHD (1991). Intraspecific variation in population gene diversity and effective population size correlates with mating systems in plants. Proc. Natl. Acad. Sci. USA.

[65] Yan JJ, Bai SQ, Zhang XQ, Chang D (2010). Genetic diversity of native *Elymus sibiricus* populations in the Southeastern Margin of Qinghai-Tibetan Plateau as detected by SRAP and SSR marker. Acta Pratacult. Sin..

[66] Aros D, Meneses C, Infante R (2006). Genetic diversity of wild species and cultivated varieties of alstroemeria estimated through morphological descriptors and RAPD markers. Sci. Hortic..

[67] Souframanien J, Gopalakrishna T (2004). A comparative analysis of genetic diversity in black gram genotypes using RAPD and ISSR markers. Theor. Appl. Genet..

[68] Gorji AM, Poczai P, Polgar Z, Taller J (2011). Efficiency of arbitrarily amplified dominant markers (SCoT, ISSR and RAPD) for diagnostic fingerprinting in tetraploid potato. Am. J. Potato Res..

[69] Saxena S (2005). Analysis of genetic diversity among papaya cultivars using single primer amplification reaction (SPAR) methods. J. Hortic. Sci. Biotech..

[70] Murty SG (2013). Comparison of RAPD, ISSR and DAMD markers for genetic diversity assessment between accessions of *Jatropha curcas* L., and its related species. J. Agric. Sci Tech..

[71] Ferrao LFV (2013). Comparative study of different molecular markers for classifying and establishing genetic relationships in *Coffea canephora*. Plant. Syst. Evol..

[72] Doyle JJ, Doyle JL (1987). A rapid DNA isolation procedure for small quantities of fresh leaf tissue. Phytochem. Bull..

[73] Doyle JJ, Doyle JL (1990). Isolation of plant DNA from fresh tissue. Focus.

[74] Sambrook, J., Fritsch, E. F. & Maniatis, T., Agarose gel electrophoresis of DNA and pulse field gel electrophoresis. In: *Molecular Cloning: a Laboratory Manual*, 3rd Edn. Cold Springer Harbor Laboratory Press, (pp. 5.1–5.86). New York, USA (1989).

[75] Zhou Z, Bebeli PJ, Somers DJ, Gustafson JP (1997). Direct amplification of minisatellite-region DNA with VNTR core sequences in the genus Oryza. Theor. Appl. Genet..

[76] Winberg BC, Shori Z, Dallas JF, Mclntyre CL, Gustafson JP (1993). Characterization of minisatellite sequences from *Oryza sativa*. Genome.

[77] Kang HW, Park DS, Go SJ, Eun MY (2002). Fingerprinting of diverse genomes using PCR with universal rice primers generated from repetitive sequence of Korean weedy rice. Mol. Cell..

[78] Jeffreys AJ, Wilson V, Thein SL (1985). Hypervariable minisatellite regions in human DNA. Nature.

[79] Nakamura Y (1987). Variable number of tandem repeats (VNTR) markers for human gene mapping. Science.

[80] Anderson TH, Nilsson-Tillgren T (1997). A fungal minisatellite. Nature.

[81] Collard BCY, Mackill DJ (2009). Start Codon Targeted (SCoT) polymorphism: a simple novel DNA marker technique for generating gene-targeted markers in plants. Plant. Mol. Biol. Rep..

[82] Luo C, He XH, Chen H, Ou SJ, Gao MP (2010). Analysis of diversity and relationships among mango cultivars using start codon targeted (SCoT) markers. Biochem. Syst. Ecol..

[83] Singh AK (2013). CAAT box-derived polymorphism (CBDP): A novel promoter-targeted molecular marker for plants. J. Plant Biochem. Biotech..

[84] Schnell RJ, Olano CT, Quintanilla WE, Meerow AW (2005). Isolation and characterization of 15 microsatellite loci from mango (*Mangifera indica* L.) and cross-species amplification in closely related taxa. Mol. Ecol. Notes..

[85] Viruel MA, Escribano P, Barbieri M, Ferri M, Hormaza JI (2005). Fingerprint, embryo type, and geographic differentiation in mango (*Mangifera indica* L., Anacardiaceae) with microsatellites. Mol. Breed..

[86] Ukoskit K (2007). Development of microsatellite markers in mango (*Mangifera indica* L.) using 5' anchored PCR. Thammasat. Int. J. Sci. Tech..

[87] Ravishankar KV, Mani BHR, Anand L, Dinesh MR (2011). Development of new microsatellite markers from mango (*Mangifera indica*) and cross-species amplification. Am. J. Bot..

[88] Yeh, F.C., Yang, R.C. & Boyle, T., POPGENE Version 1.32: Microsoft Window-Based Freeware for Population Genetics Analysis, (p. 12). University of Alberta, Edmonton (1999).

[89] Peakall R, Smouse PE (2012). GenAlEx 6.5: Genetic analysis in Excel. Population genetic software for teaching and research-an update. Bioinformatics.

[90] Jaccard P (1908). Nouvellesrecherchessur la distribution florale. Bull. Soc. vaudoise sci. nat..

[91] Rohlf, F.J. NTSYS pc numerical taxonomy and multivariate system, Version 2.1.Exeter Publ Ltd, Setauket, New York (1993).

[92] Sneath PHA, Sokal RR (1973). Numerical taxonomy.

[93] Nei M (1972). Genetic distance between populations. Am. Nat..

[94] Yap, V., Nelson, R. J. WinBoot: A program for performing bootstrap analysis of binary data to determine the confidence limits of UPGMA-based dendrograms. IRRI, Philippines (1996).

