# Supplementary material for: Multiple DNA marker-assisted diversity analysis of Indian mango (Mangifera indica L.) populations
Source: Sci Rep. 2021 May 14;11:10345. doi: 10.1038/s41598-021-89470-3 (PMC8121829; doi:10.1038/s41598-021-89470-3)
Supplement: Supplementary file 1 — Supplementary Information. [file 41598_2021_89470_MOESM1_ESM.pdf]

Multiple DNA Marker-Assisted Diversity Analysis of Indian Mango (*Mangifera indica* L.) Populations

Ram Chandra Jena and Pradeep Kumar Chand[†]

Plant Biotechnology Laboratory, Post-Graduate Department of Botany, Utkal University, Bhubaneswar -751004, Odisha, India

† **Corresponding author :** Dr. Pradeep Kumar Chand, e-mail: pkchand13@gmail.com

Suppl. Table S1. AMOVA analysis for 4 Geographic Populations (East India, West India, North India, South India) of selected Indian mangoes based on individual and cumulative DNA marker systems.

Marker (s)	Source of variance	df	SS	MS	Est. Var.	Tot. Var. (%)	Φ_{PT}	*p* Value *
**RAPD**	Among populations	3	149.83	49.94	2.14	9		
	Within population	54	1234.38	22.86	22.86	91		
	Total	57	1384.21		25.00	100	0.086	0.000
**ISSR**	Among populations	3	17.90	5.97	0.18	5		
	Within population	54	197.84	3.66	3.66	95		
	Total	57	215.74		3.85	100	0.047	0.006
**DAMD**	Among populations	3	257.56	85.86	4.26	12		
	Within population	54	1733.82	32.11	32.11	88		
	Total	57	1991.38		36.36	100	0.117	0.000
**SCoT**	Among populations	3	40.59	13.53	0.30	3		
	Within population	54	528.79	9.79	9.79	97		
	Total	57	569.38		10.09	100	0.029	0.009
**CBDP**	Among populations	3	92.97	30.99	0.87	4		
	Within population	54	1080.05	20.00	20.00	96		
	Total	57	1173.02		20.87	100	0.042	0.001
**SSR**	Among populations	3	294.50	98.17	5.12	13		
	Within population	54	1811.90	33.55	33.55	87		
	Total	57	2106.40		38.67	100	0.122	0.000
**RAPD+ISSR +DAMD**	Among populations	3	434.20	144.73	6.63	10		
	Within population	54	3293.84	61.00	61.00	90		
	Total	57	3728.03		67.63	100	0.098	0.000
**SCoT+CBDP**	Among populations	3	133.55	44.52	1.17	4		
	Within population	54	1608.84	29.79	29.79	96		
	Total	57	1742.40		30.96	100	0.038	0.001

df: *degree of freedom,* ***SS:*** *sum of squares,* ***MS:*** *mean of squares,* ***Est.*** *Var.***:** *estimated variance,* ***Tot. Var. (%):*** *percentage of total variance;* Φ_{PT}**:** *coefficient of genetic differentiation. * Significant test after 9999 random permutations.*

Suppl. Table S2. AMOVA analysis for 3 Fruit Status Populations (Selection, Hybrid, Landrace) of selected Indian mangoes based on individual and cumulative DNA marker systems.

Marker (s)	Source of variance	df	SS	MS	Est. Var.	Tot. Var. (%)	Φ_{PT}	*p* Value *
**RAPD**	Among populations	2	181.90	90.95	3.01	11		
	Within population	55	1809.48	32.90	32.90	89		
	Total	57	1991.38		35.91	100	0.084	0.000
**ISSR**	Among populations	2	84.23	42.11	0.96	5		
	Within population	55	1299.98	23.64	23.64	95		
	Total	57	1384.21		24.60	100	0.052	0.001
**DAMD**	Among populations	2	22.18	11.09	0.26	4		
	Within population	55	330.27	6.01	6.01	96		
	Total	57	352.45		6.27	100	0.042	0.000
**SCoT**	Among populations	2	29.96	14.98	0.27	3		
	Within population	55	539.42	9.81	9.81	97		
	Total	57	569.38		10.08	100	0.027	0.004
**CBDP**	Among populations	2	62.99	31.50	0.59	3		
	Within population	55	1110.03	20.18	20.18	97		
	Total	57	1173.02		20.77	100	0.028	0.004
**SSR**	Among populations	2	14.96	7.48	0.20	4		
	Within population	55	200.78	3.65	3.65	96		
	Total	57	215.74		3.85	100	0.039	0.000
**RAPD+ISSR+ DAMD**	Among populations	2	288.31	144.15	4.24	6		
	Within population	55	3439.73	62.54	62.54	94		
	Total	57	3728.03		66.78	100	0.063	0.000
**SCoT+CBDP**	Among populations	2	92.95	46.48	0.86	3		
	Within population	55	1649.45	29.99	29.99	97		
	Total	57	1742.40		30.85	100	0.028	0.002

df: *degree of freedom,* ***SS:*** *sum of squares,* ***MS:*** *mean of squares,* **Est.** *Var.***:** *estimated variance,* ***Tot. Var. (%):*** *percentage of total variance;* Φ_{PT}**:** *coefficient of genetic differentiation. * Significant test after 9999 random permutations.*

Suppl. Table S3. Matrix of unbiased genetic identity and distance among 4 Geographical Populations of selected Indian mangoes based on cumulative Arbitrary (RAPD + ISSR + DAMD), cumulative Gene targeted (SCoT + CBDP) and SSR marker systems.

RAPD + ISSR+DAMD				
Population	EI	WI	NI	SI
EI	****	0.83	0.90	0.93
WI	0.17	****	0.90	0.88
NI	0.10	0.10	****	0.91
SI	0.07	0.12	0.09	****
SCoT + CBDP				
Population	EI	WI	NI	SI
EI	****	0.91	0.92	0.95
WI	0.09	****	0.91	0.90
NI	0.08	0.09	****	0.93
SI	0.05	0.10	0.07	****
SSR				
Population	EI	WI	NI	SI
EI	****	0.81	0.86	0.94
WI	0.19	****	0.91	0.88
NI	0.14	0.09	****	0.85
SI	0.06	0.12	0.15	****

*Nei's (1972) genetic identity (above diagonal) and genetic distance (below diagonal).Cells with **** are for the same populations. EI: East India, WI: West India; NI: North India; SI: South India*

Suppl. Table S4. Matrix of unbiased genetic identity and distance among 3 Fruit Status Populations of selected Indian mangoes based on cumulative Arbitrary (RAPD + ISSR + DAMD), cumulative Gene targeted (SCoT + CBDP) and SSR marker systems.

**RAPD + ISSR + DAMD**			
Population	S	H	L
S	****	0.96	0.91
H	0.04	****	0.96
L	0.09	0.04	****
**SCoT + CBDP**			
Population	S	H	L
S	****	0.97	0.94
H	0.03	****	0.94
L	0.06	0.06	****
**SSR**			
Population	S	H	L
S	****	0.96	0.92
H	0.04	****	0.92
L	0.08	0.08	****

*Nei's (1972) genetic identity (above diagonal) and genetic distance (below diagonal).Cells with **** are for the same populations. S: Selection, H: Hybrid and L: Landrace*

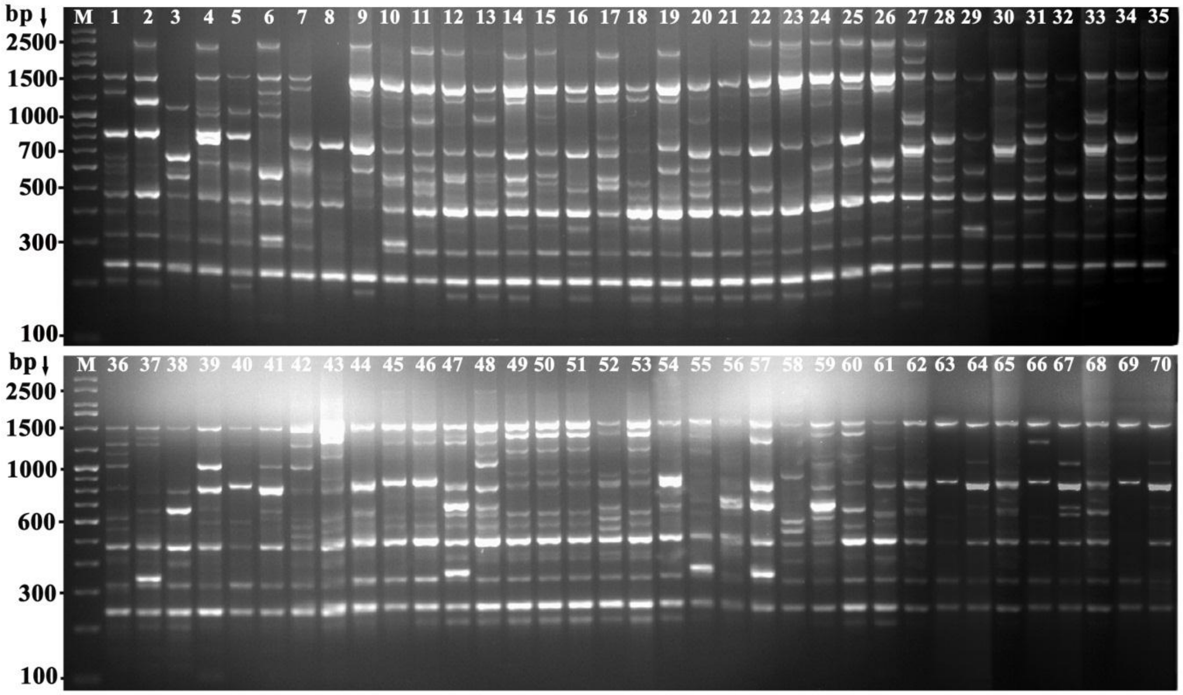

Suppl. Fig. 1a. RAPD profiling of 70 selected Indian mango genotypes using primer OPA 18. Lane M: Low Range DNA Ruler Plus. Lanes 1-70 correspond to the mango genotypes (Listed in Table 1).

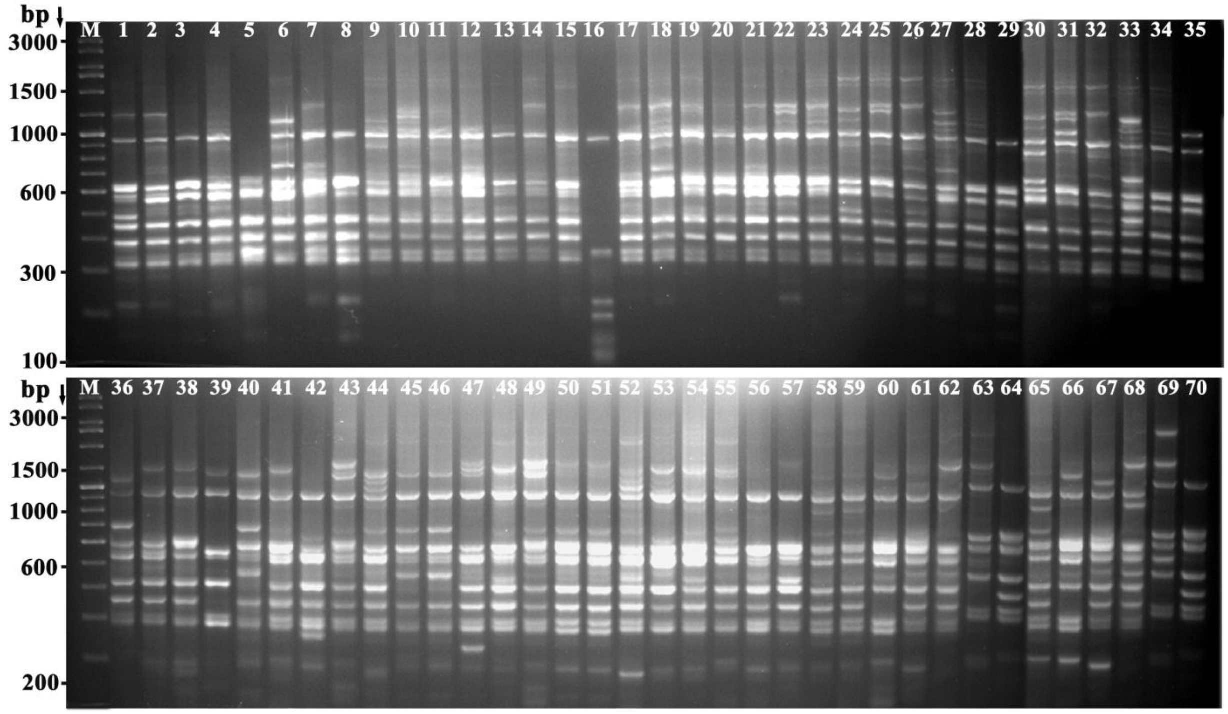

Suppl. Fig. 1b. ISSR profiling of 70 selected Indian mango genotypes using primer ISSR-9. Lane M: Low Range DNA Ruler Plus. Lanes 1-70 correspond to the mango genotypes (Listed in Table 1).

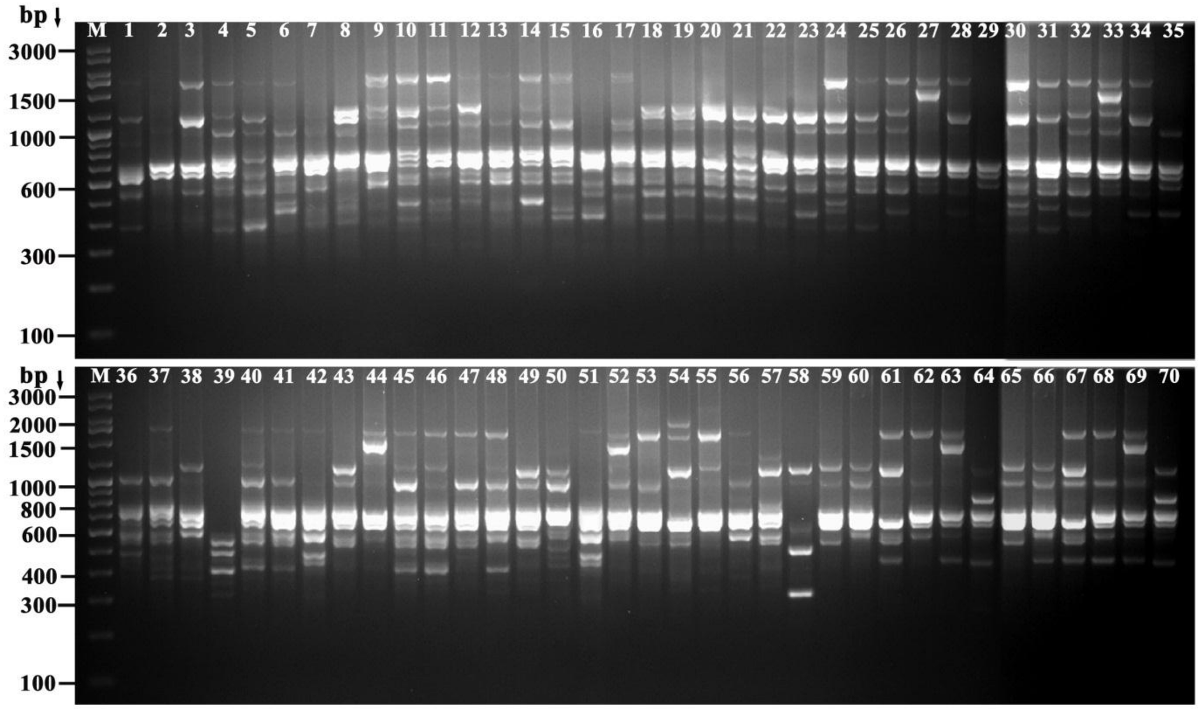

Suppl. Fig. 1c. DAMD profiling of 70 selected Indian mango genotypes using primer HBV. Lane M: Low Range DNA Ruler Plus. Lanes 1-70 correspond to the mango genotypes (Listed in Table 1).

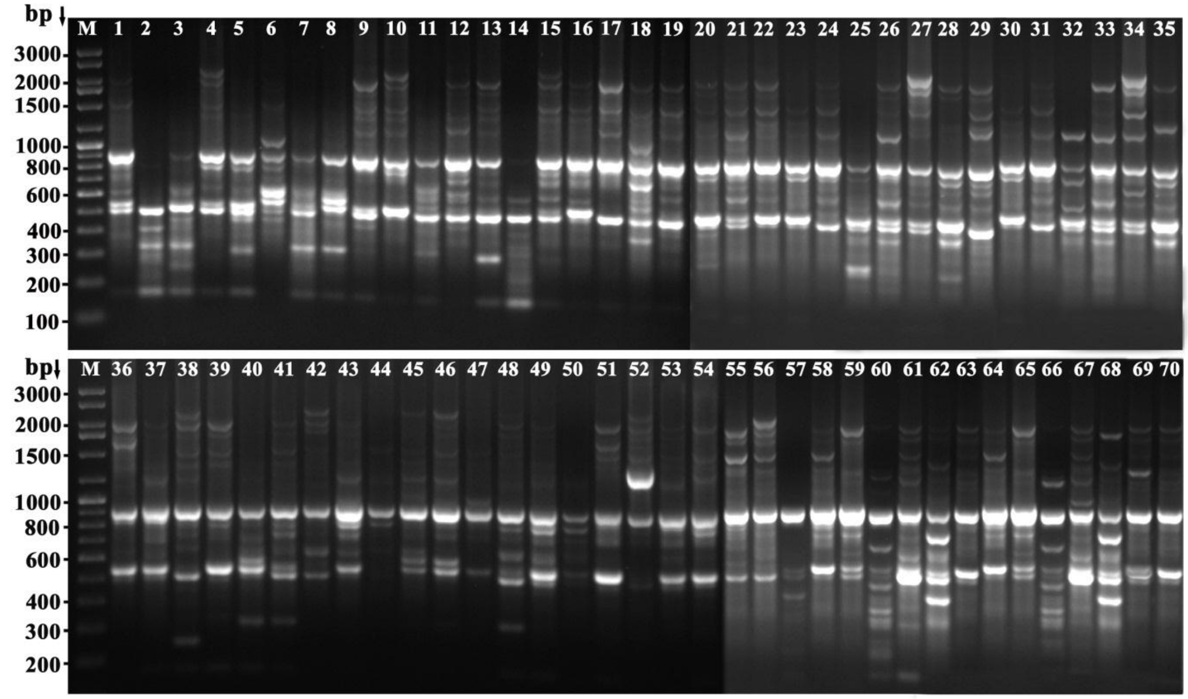

Suppl. Fig. 1d. SCoT profiling of 70 selected Indian mango genotypes using primer SCoT 8. Lane M: Low Range DNA Ruler Plus. Lanes 1-70 correspond to the mango genotypes (Listed in Table 1).

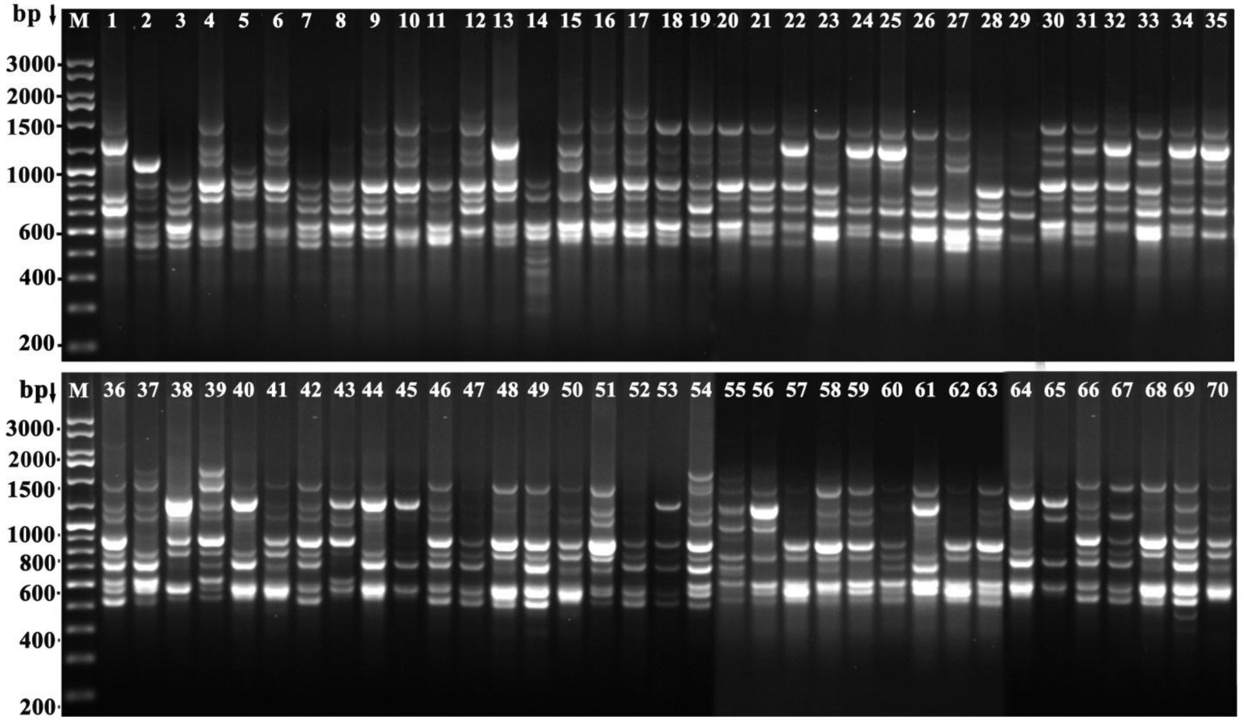

Suppl. Fig. 1e. CBDP profiling of 70 selected Indian mango genotypes using primer CAAT-3. Lane M: Low Range DNA Ruler Plus. Lanes 1-70 correspond to the mango genotypes (Listed in Table 1).

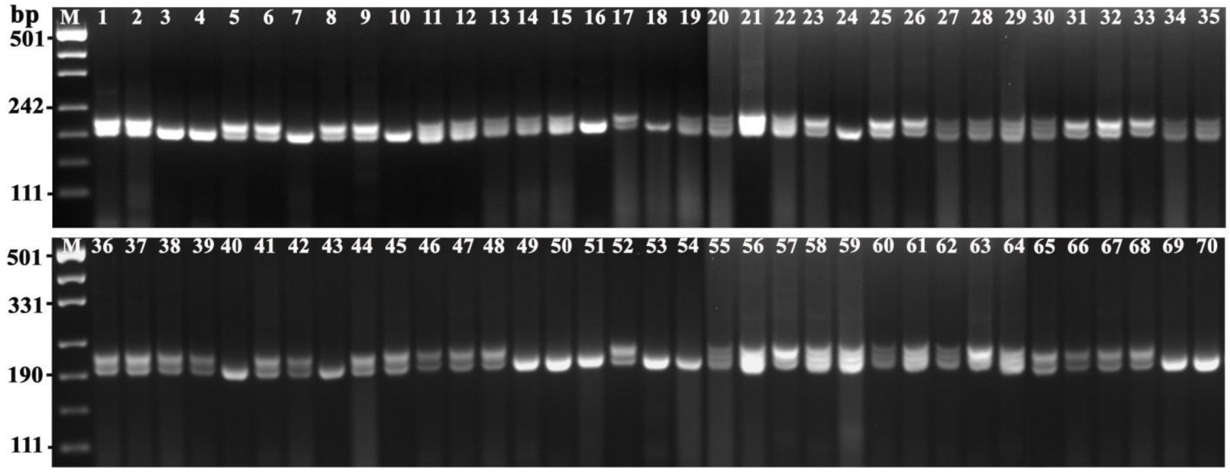

Suppl. Fig. 1f . SSR profiling of 70 selected Indian mango genotypes using primer SSR-20. Lane M: pUC19/DNA Msp I digest. Lanes 1-70 correspond to the mango genotypes (Listed in Table 1).

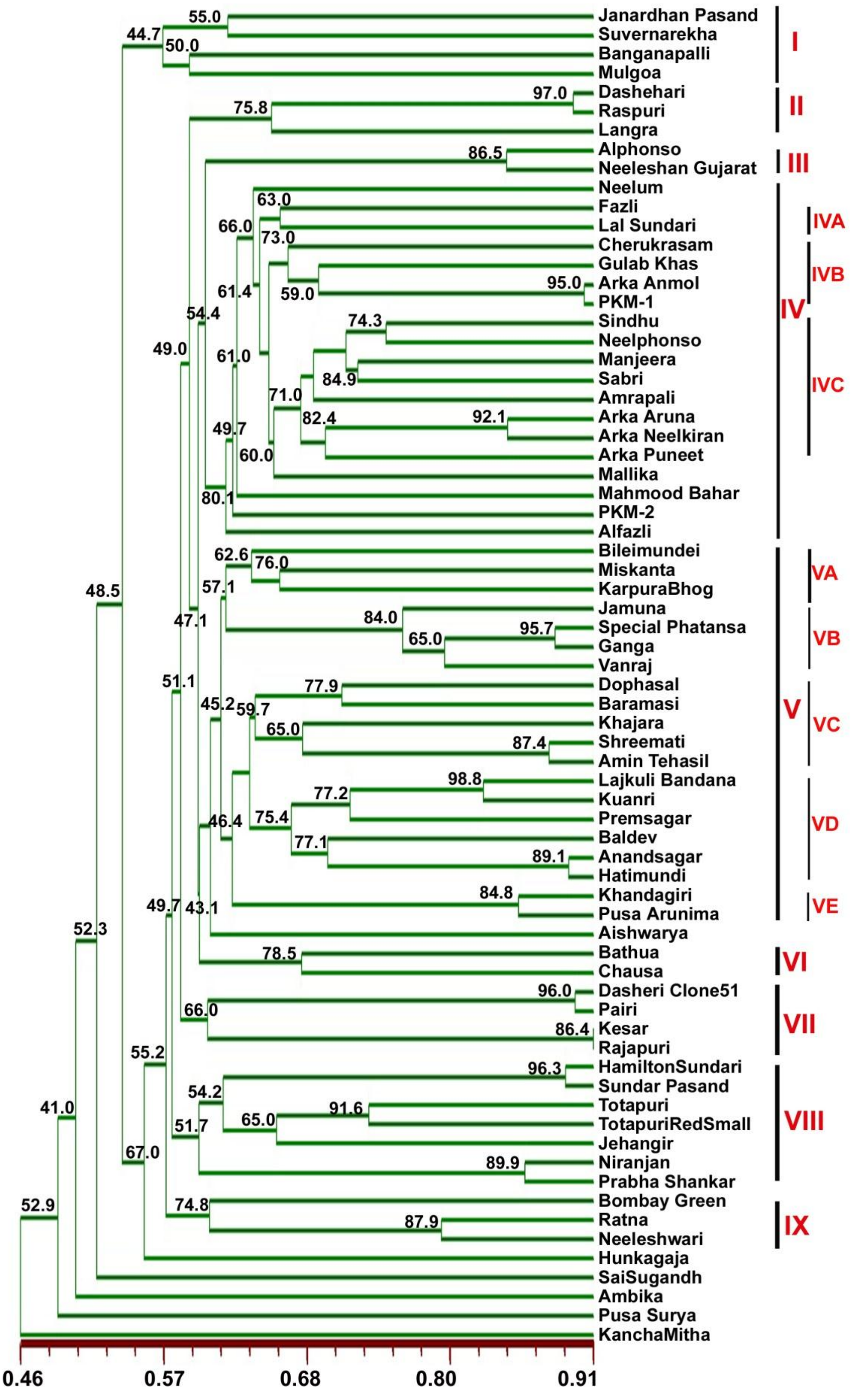

Suppl. Fig. 2a: Dendrogram of 70 Indian mango genotypes constructed based on cumulative RAPD+ISSR+DAMD+SCoT+CBDP+SSR markers using UPGMA method. (*Numbers indicate mango genotypes as listed in Table 1, Values at the nodes correspond to bootstrap support [1000 replications])*

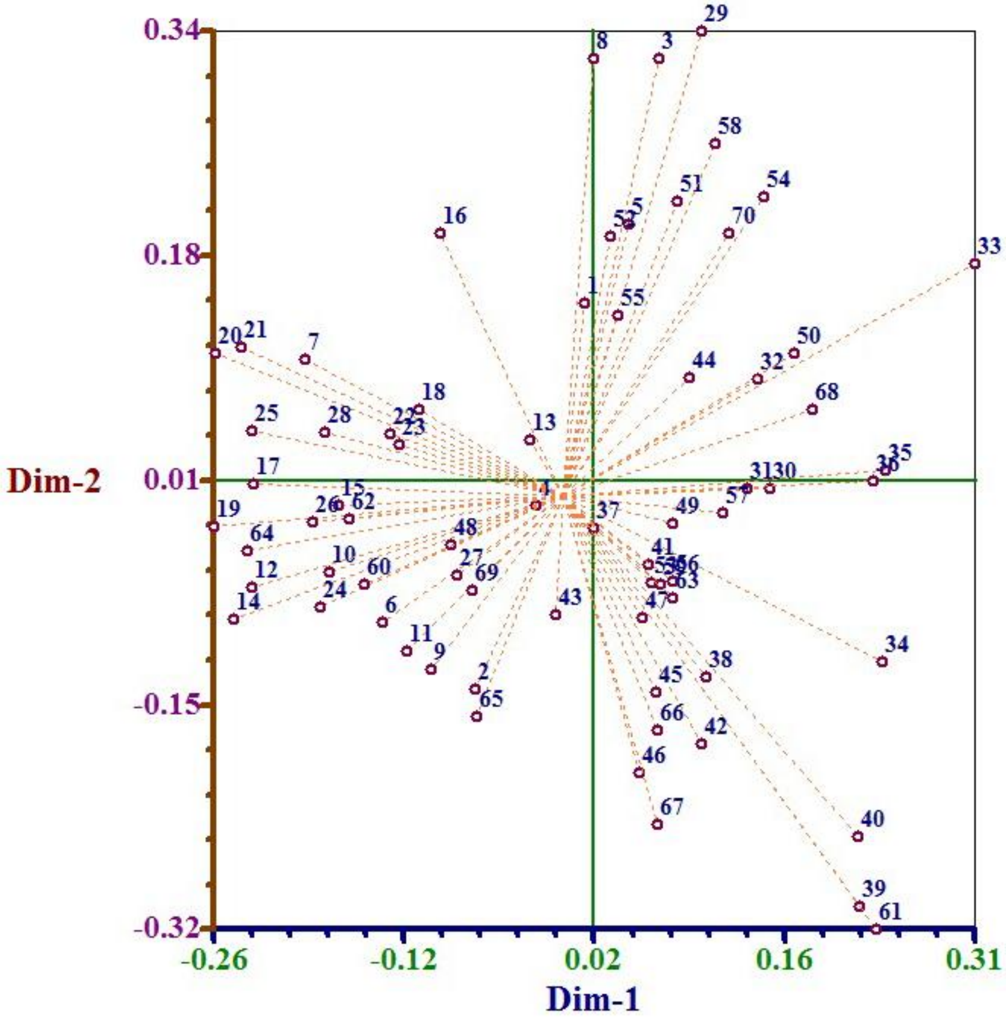

Suppl. Fig. 2b: Two dimensional distribution of 70 selected Indian mango genotypes by PCA using cumulative RAPD+ISSR+DAMD+SCoT+CBDP+SSR markers. Numbers plotted (1-70) represent individual genotypes as listed in Table 1

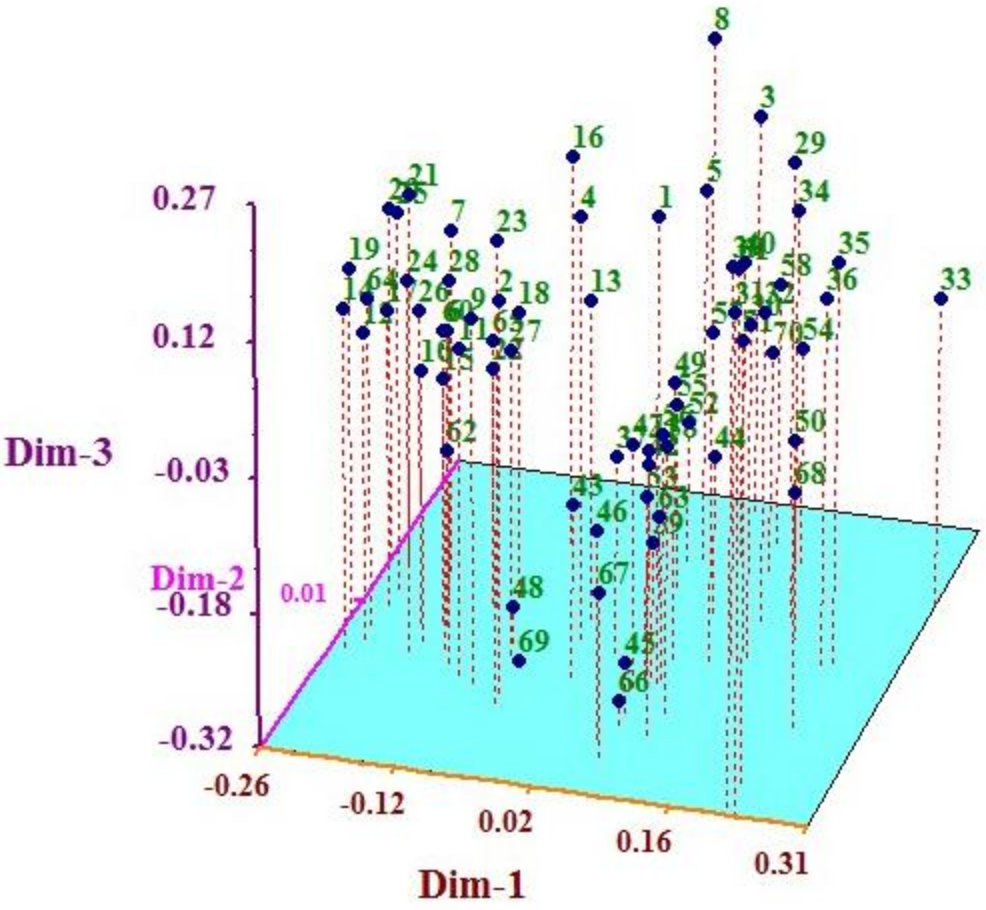

Suppl. Fig. 2c: Three dimensional distribution of 70 selected Indian mango genotypes revealed by PCA analysis based on cumulative RAPD+ISSR+DAMD+SCoT+CBDP+SSR markers data. Numbers plotted (1-70) represent individual genotypes as listed in Table 1
